# 3,4-Ethylenedioxythiophene Hydrogels: Relating Structure and Charge Transport in Supramolecular Gels

**DOI:** 10.1021/acs.chemmater.3c01360

**Published:** 2024-03-25

**Authors:** Luke C. B. Salter, Jonathan P. Wojciechowski, Ben McLean, Patrick Charchar, Piers R. F. Barnes, Adam Creamer, James Doutch, Hanna M. G. Barriga, Margaret N. Holme, Irene Yarovsky, Molly M. Stevens

**Affiliations:** † Department of Materials and Department of Bioengineering, Institute of Biomedical Engineering, Imperial College London, London SW7 2AZ, United Kingdom; ‡ School of Engineering, RMIT University, Melbourne, Victoria 3001, Australia; § Department of Physics, Imperial College London, London SW7 2AZ, United Kingdom; ∥ ISIS Muon and Neutron Source, Rutherford Appleton Laboratory, Harwell Campus, Oxfordshire OX11 0QX, United Kingdom; ⊥ Department of Medical Biochemistry and Biophysics, Karolinska Institute, 171 77 Stockholm, Sweden; △ ARC Research Hub for Australian Steel Innovation, https://www.rmit.edu.au/research/centres-collaborations/multi-partner-collaborations/arc-research-hub-aus-steel-manufacturing; ▽ Department of Physiology, Anatomy and Genetics, Department of Engineering Science, and Kavli Institute for Nanoscience Discovery, University of Oxford, OX1 3QU, Oxford, United Kingdom

## Abstract

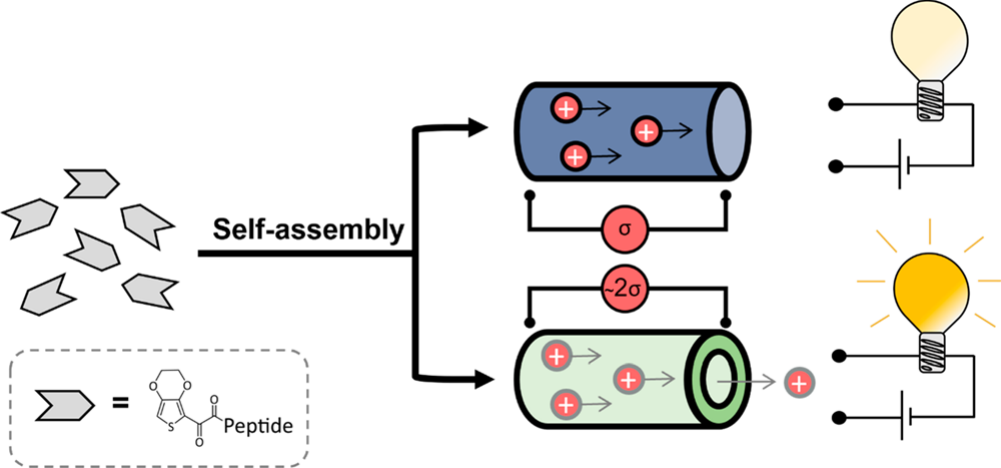

Ionic charge transport is a ubiquitous language of communication in biological systems. As such, bioengineering is in constant need of innovative, soft, and biocompatible materials that facilitate ionic conduction. Low molecular weight gelators (LMWGs) are complex self-assembled materials that have received increasing attention in recent years. Beyond their biocompatible, self-healing, and stimuli responsive facets, LMWGs can be viewed as a “solid” electrolyte solution. In this work, we investigate 3,4-ethylenedioxythiophene (EDOT) as a capping group for a small peptide library, which we use as a system to understand the relationship between modes of assembly and charge transport in supramolecular gels. Through a combination of techniques including small-angle neutron scattering (SANS), NMR-based Van’t Hoff analysis, atomic force microscopy (AFM), rheology, four-point probe, and electrochemical impedance spectroscopy (EIS), we found that modifications to the peptide sequence result in distinct assembly pathways, thermodynamic parameters, mechanical properties, and ionic conductivities. Four-point probe conductivity measurements and electrochemical impedance spectroscopy suggest that ionic conductivity is approximately doubled by programmable gel assemblies with hollow cylinder morphologies relative to gels containing solid fibers or a control electrolyte. More broadly, it is hoped this work will serve as a platform for those working on charge transport of aqueous soft materials in general.

## Introduction

Self-assembled systems have the potential to recapitulate the microstructural complexity and self-monitoring of biological systems for the development of novel biomaterials. Within this expanding field, subtle incorporation of conductivity into soft matter such as hydrogels is a persistent goal given that electrical stimulation can enhance differentiation and proliferation in relevant tissue such as those containing cardiac and nerve cells.^1−5^ When exploring the electrical stimulation of cells, it is particularly important to consider the ionic transport potential of the gels, since biological systems communicate primarily in this form of charge movement. Therefore, while developing materials to enhance electronic transport in soft materials is valuable (e.g., through conductive polymer incorporation), the field must also innovate methods and materials to study and enhance ionic charge flow.

To have meaningful control over the properties of a material, it can be preferable to operate with those that are, as in nature, well-defined across multiple length scales over random, heterogeneous systems. In this vein, supramolecular materials offer superiority through the self-optimizing and “self-checking” capacity built into their design. Most relevant to bioengineering applications are self-assembled hydrogels formed from low molecular weight gelators (LMWGs), a class of soft materials that have gained increasing attention in recent years.^6−11^ A common subsection of these materials utilizes short peptide sequences engineered to self-assemble into fiber morphologies which entangle, encapsulating water to form a hydrogel. The subsequent materials not only offer potential for chemical modularity but also demonstrate reversibility between gel and solution states due to the noncovalent interactions which cross-link the materials. We have been interested in these dynamic and responsive systems as their fibrous networks serve as excellent mimics for biological tissue both morphologically and mechanically. Though many examples have sought to explore the addition of conjugated oligomers to induce charge transfer events intramolecularly,^12−14^ few have examined charge transport, both electronic and ionic, through the macrostructure as a whole. Examples that do explore conductivity in assembled conjugated compounds generally focus on organic soluble systems with many characterized as dried films.^15−18^ We note that previous work exists, such as from Hochbaum and co-workers,^19^ examining particularly electronic charge transport along nanotubes formed of long peptide sequences, but we emphasize that here we focus on ionic contributions within the hydrogel bulk as a whole. Further, other conductive hydrogel studies broadly ignore interrelations between assembly, structure, and charge transport with authors often opting to polymerize post-assembly transitioning away from a truly supramolecular architecture.^20,21^

In this work we set out to incorporate the 3,4-ethylenedioxythiophene (EDOT) moiety into a LMWG system for use in the field of bioelectronics and to probe the relation between design, assembly, and charge transport in supramolecular gels. We have broadly investigated the charge transport properties achieved by incorporating EDOT oligomers into low molecular weight gelators. In developing this library, we were also interested in elucidating the ionic contributions and, as such, investigated gelators capped with only a single EDOT unit. Though this short conjugation length would clearly not give electronic conductivities or the contribution would likely be small, it was seen as a suitable system to interrogate specifically the ionic charge transport contributions and their structural origin. Herein, we report on the assembly of EDOT-capped peptides into both elliptical and hollow cylindrical fibers, as determined by small-angle neutron scattering. We propose that the difference in assembly is primarily driven by hydrophobicity and rigidity (or lack thereof) of the selected peptide sequence and that the apparent directional bonding of the EDOT-FFF hollow fibers might be enforced by stacking of a more H-like, face-to-face aggregation type. We speculate that while the nature of assembly in the elliptical fibers is likely following the established spherical to worm-like micelle transition,^22^ the hollow fibers of EDOT-FFF adopt fiber morphologies even before the gelation trigger. We then proceed to relate this to thermodynamic parameters of the assembled systems, finding that as expected, greater enthalpy and entropy are released upon dissolution of the more hydrophobic and less flexible EDOT-FFF, hollow fiber example. We then analyze the system by molecular dynamics (MD) simulations which suggested EDOT-FFF interfaced primarily via π–π interactions and shielded its backbone from solvent, preventing hydrogen bonding, while EDOT-GFF and EDOT-GFFD incorporated a combination of hydrogen bonding and π–π interactions with the possibility of β sheet formation, particularly in the latter. We finally fully characterize the ionic charge transport capacity of the systems and note a substantially greater value for the hollow cylinders of EDOT-FFF even when compared to the electrolyte without a gelator. We anticipate that this work will serve as a starting point and roadmap to understanding structure–charge transport characterization in self-assembled hydrogels and more broadly in understanding charge diffusion of aqueous soft matter generally.

**Figure 1:**
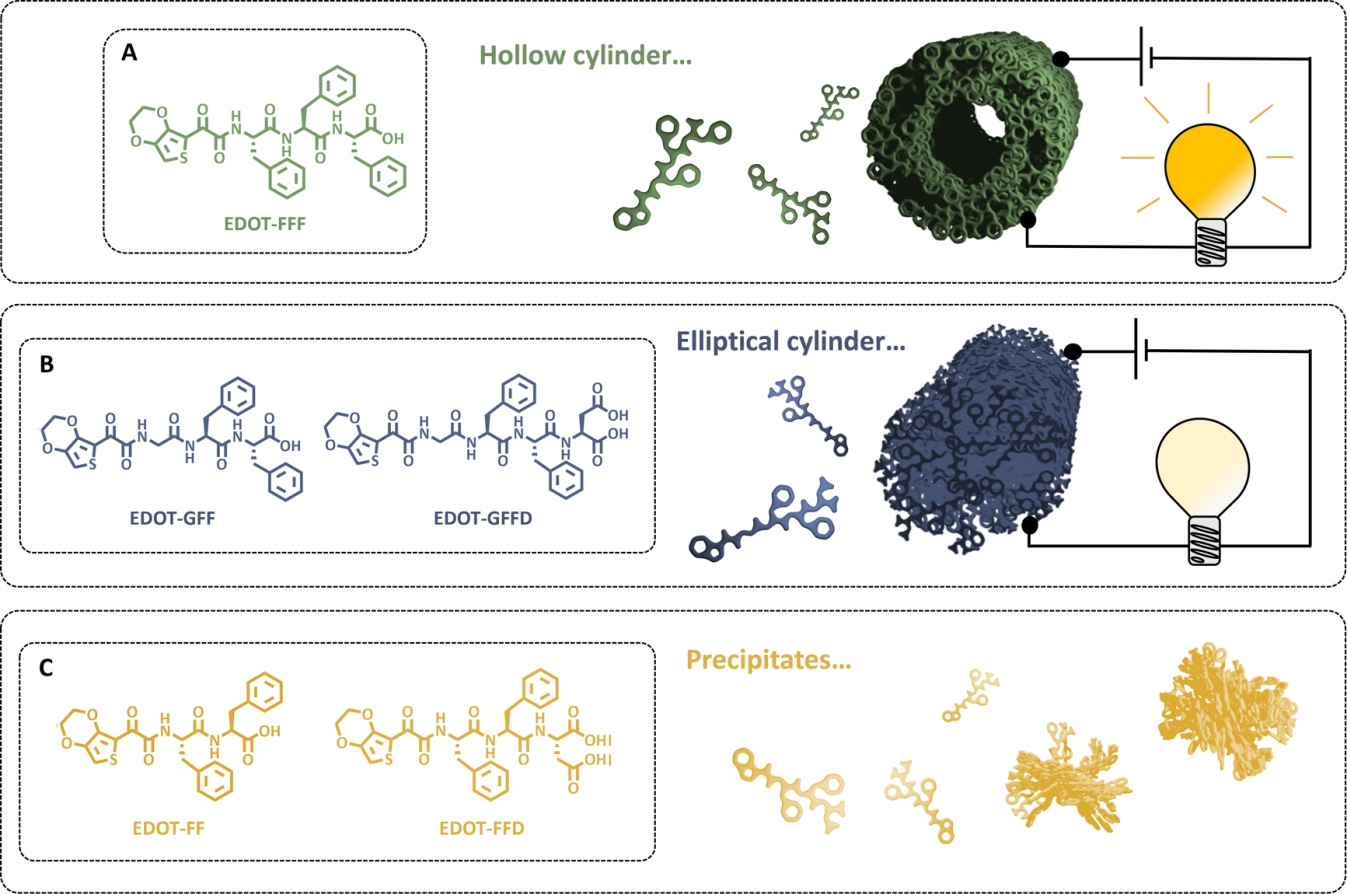
An illustration of the three categories of self-assembly observed in the five peptides tested labeled A–C. It was seen EDOT-FFF formed hollow fibers (A) with greater conductivity and EDOT-GFF and EDOT-GFFD formed elliptical fibers (B) with less conductivity, while EDOT-FFD and EDOT-FF simply precipitated (C). Note the schematics shown here are artistic approximations and not modeled data. All include an EDOT capping group coupled to a sequence containing at least two l-phenylalanine units.

To incorporate an EDOT moiety into the self-assembled hydrogels, five peptide sequences were selected with their N-terminus modified with 3,4-ethylenedioxythiophene-2-oxalic acid (Figure 1).

## Results and Discussion

### Peptide Design and Hydrogel Formation

Our peptide sequence selections were based on probing varying degrees of hydrophobicity while still forming supramolecular hydrogels. All the peptides were based around the inclusion of at least two adjacent l-phenylalanine (Phe, F) amino acids as this has been shown to promote the formation of fibrous morphologies and increase hydrophobicity.^23^ Previous work based on coarse-grained molecular dynamics of the tripeptide design space related the chance of gelation to a parameter defined as aggregation propensity (AP), a variable that correlated with hydrophobicity making phenylalanine a leading choice when attempting to design molecular gelators.^24^

Glycine (Gly, G) and l-aspartic acid (Asp, D) amino acids were chosen to investigate the role of greater flexibility of the EDOT capping group and to modulate the hydrophobicity of the N-functionalized peptides. Glycine can act as a flexible spacer between the N-terminal capping group and peptide sequence while the presence of two carboxyl groups in aspartic acid lends greater hydrophilicity to the molecule potentially delaying its desolvation when the pH is lowered to trigger gel formation.^25^ Initially, FFF was chosen to maximize hydrophobicity along with FFD, a commonly used sequence of greater hydrophilicity that still contains the diphenylalanine motif.^26^ Expanding from here, we incorporated the glycine linker flexibility to select GFF and GFFD along with simply FF itself given the literature precedent already mentioned.

Functionalization of EDOT with an oxoacetic acid group, **2** (Figure S1), was readily achieved by chemistry previously developed in our group via glyoxylation of EDOT with oxalyl chloride, followed by esterification with methanol to synthesize the methyl oxalate ester derivative, **1**, which was hydrolyzed under basic conditions to the oxoacetic acid derivative, **2**, with a yield of 64% over two steps (Figure S1).^27^

Conventional fluorenemethoxycarbonyl (Fmoc)-based solid phase synthesis methods were employed to afford the desired N-functionalized peptide library with purities > 95% (see SI for additional experimental details). Gelation of the hydrogels was triggered by the slow hydrolysis of glucono-δ-lactone (GdL) to gluconic acid, pioneered in the Adams lab.^28^ This trigger allows for a slow decrease in pH that gradually protonates the compounds, reducing their solubility in aqueous solvents and initiating the self-assembly of the peptides into fibrous structures to form hydrogels. Gelation is achieved through the entanglement of these fibers creating a dense matrix that in turn traps water molecules. This method is thought to be the most reliable and reproducible in the material class.^22^ The peptides were initially solubilized in a basic environment (∼pH 11–12) and then mixed with GdL. The exact final pH after GdL hydrolysis will vary depending on the p*K*_a_ (or apparent p*K*_a_) of the assemblies/aggregates but will often be in the 3–5 pH range.^26^ Upon mixing with GdL, it was found that EDOT-FF and EDOT-FFD simply precipitated (pathway **C** of Figure 1). While these sequences have been shown to form hydrogels in other related N-terminal aromatic capped systems,^26,29^ no ordered assembly could be achieved when using EDOT oxoacetic as an N-terminal group, illustrating the inherent uncertainty met with when designing sequences for LMWGs and demonstrates the effect of capping group identity.^30^ Preliminary vial inversion tests demonstrated EDOT-FFF, EDOT-GFF, and EDOT-GFFD (Figure S2) could support their weight to concentrations as low as 1–2 mM. These could further be delineated into two further pathways of assembly (pathways **A** and **B** of Figure 1) to be discussed below.

**Figure 2:**
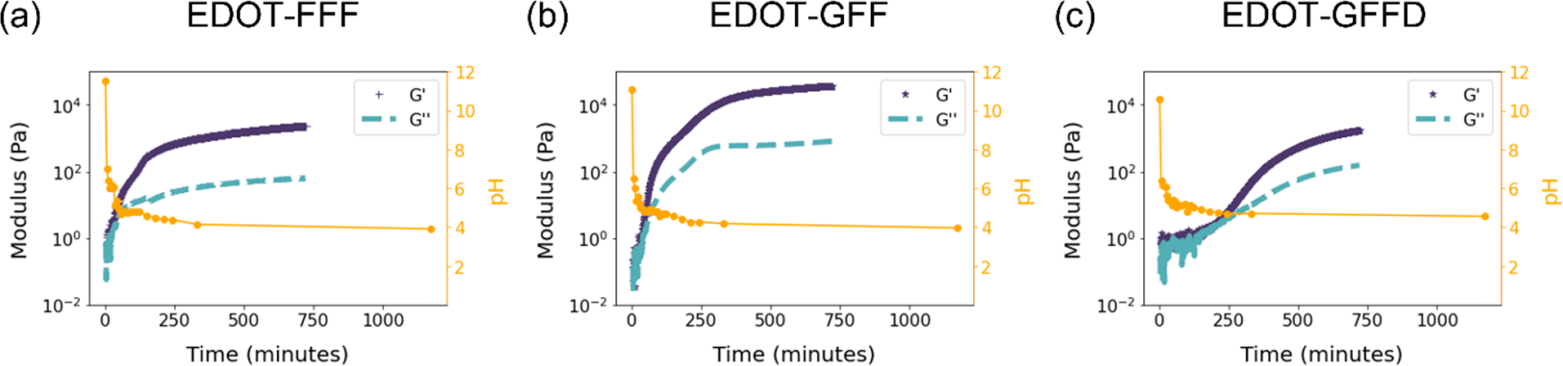
Time-resolved rheology and pH data (in orange) for (a) EDOT-FFF, (b) EDOT-GFF, and (c) EDOT-GFFD. Rheology parameters: concentration = 10 mM, frequency = 0.1 Hz, strain = 0.1%.

To confirm that EDOT-FFF, EDOT-GFF, and EDOT-GFFD had formed hydrogels, rheology was used to measure the viscoelastic properties of the materials. A strain sweep from 0.01 to 100% strain showed that the EDOT-peptides had a linear viscoelastic region (LVR) up to 0.1% strain for EDOT-FFF and EDOT-GFF, whereas EDOT-GFFD showed an LVR up to 1% strain (Figure S3). Additionally, a frequency sweep showed the materials displayed a storage modulus (*G*′) ≫ than the loss modulus (*G*″) over a range of frequencies (0.01–10 Hz), affording them quantitatively as hydrogels, i.e., tan δ (*G*″/*G*′) < 0.15.^31^ To measure the gelation kinetics of the EDOT-peptides, a time-resolved sweep using a frequency of 1 Hz and strain of 0.1% was used. As shown in Figure 2, EDOT-FFF, EDOT-GFF, and EDOT-GFFD all gave an order of magnitude separation between storage and loss modulus values. Of the three EDOT-peptides, EDOT-GFF gave the highest plateau storage modulus of approximately 37 kPa. For reference, the shear moduli of biological tissue such as relaxed muscle begin from 1 kPa upward putting these gels in a suitable mechanical range as biomaterials in, for instance, cardiac repair.^32^ It was interesting to note EDOT-GFFD showed much slower gelation kinetics possibly owing to the increased solubility imparted from the two pendant carboxyl groups, which can enthalpically contribute to the solvation of the self-assembled fibers (*vide infra*) and delay their physical cross-linking. The lack of discontinuities in the data allowed for confidence that no slipping was occurring during measurement of rheology. The pH values after around 20 h were measured to be 3.91, 3.96, and 4.55 for EDOT-FFF, EDOT-GFF, and EDOT-GFFD, respectively (Figure 2). This suggests that the equilibrium of protonation is shifted further toward the aggregate than the GdL for EDOT-GFFD when compared to the other two gelators. This could be explained by the additional pendant carboxyl group offering more sites of protonation on the surface and, hence, decreasing the concentration of protons in solution. In other words, the gelator will act as a buffer against pH change; a greater number of carboxyl groups will therefore increase this buffering effect and slow the onset of gelation.

**Figure 3:**
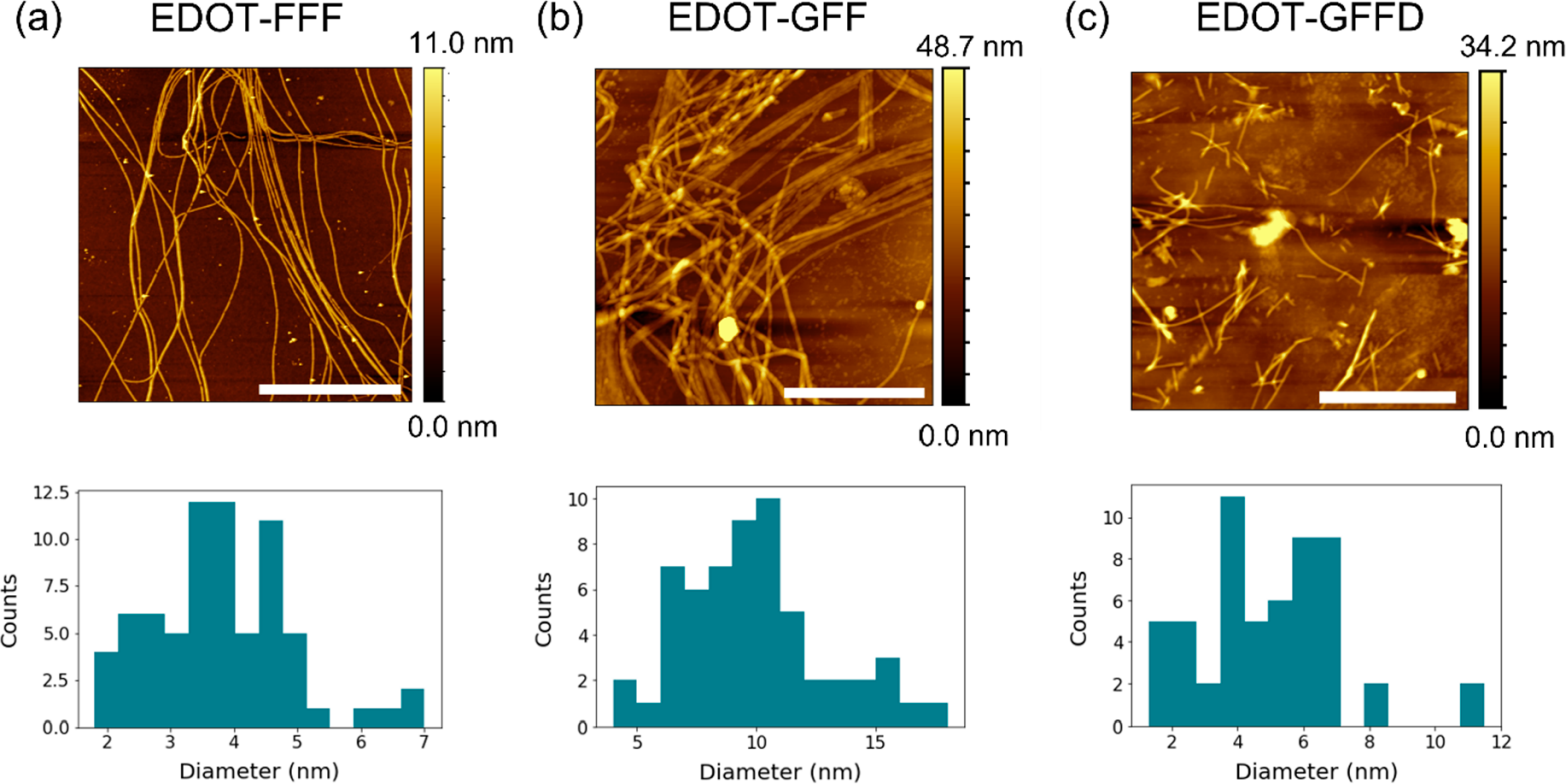
Atomic force microscopy (AFM) and height profile histograms of (a) EDOT-FFF, (b) EDOT-GFF, and (c) EDOT-GFFD. Scale bar 2 μm.

To probe the morphologies responsible for the hydrogels, atomic force microscopy (AFM) was employed on dried drop cast samples. As can be seen in Figure 3, EDOT-FFF, EDOT-GFF, and EDOT-GFFD showed entangled fiber-like morphologies consistent with that commonly seen in the literature.^11,29^ Histograms were constructed from the height profiles obtained from AFM (Figure 3). All distributions did not appear Gaussian since, in most cases, fiber bundling prevented the diameter of a single fiber from dominating statistically. Diameters down to 1.8 nm were observed for EDOT-FFF and EDOT-GFFD but only to around 4 nm in EDOT-GFF suggesting either greater predisposition for fiber bundling or different molecular arrangement.

### Probing Fiber Morphology with Neutron Scattering and UV–Visible Spectroscopy

**Figure 4:**
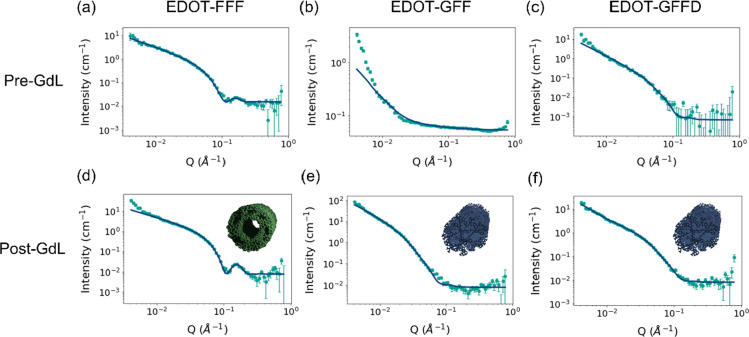
Small-angle neutron scattering (SANS) data pre- and post-GdL fitted with (a, d) a hollow cylinder model for EDOT-FFF, (b, e) a flexible elliptical cylinder model for EDOT-GFF, and (c, f) a flexible elliptical cylinder model for EDOT-GFFD.

Small-angle neutron scattering (SANS) measurements of the pre-gel solutions and hydrogels was performed in deuterated water to rule out any potential drying effects of the AFM measurements and probe the structures in a deuterated equivalent of their native environment (Figure 4, residuals in Figure S4). In the case of EDOT-FFF, it was found that a hollow cylinder model (plotted in Figure 4a) best described the high scattering wavevector Q (higher Q probes smaller length scales) features at both high and low pH. This agrees with previous work in similar systems^33^ and gives a total radius of ∼3.2 nm (Table 1) that is in agreement with the extracted height profiles from AFM (Figure 3a).

**Table 1:** Fitted SANS Parameters for the Three Gelators after Gelation

Compound	Model	Kuhn length (Å)	Inner radius (Å)	Outer radius (Å)	Axis ratio	χ^2^
EDOT-FFF	Hollow cylinder	n/a	9.21 ± 0.5	32.3 ± 3.4	n/a	3.04
EDOT-GFF	Flexible elliptical cylinder	85.5 ± 20.5	n/a	40.9 ± 2.1	2.5 ± 0.1	2.93
EDOT-GFFD	Flexible elliptical cylinder	327.6 ± 20.1	n/a	22.9 ± 0.5	2.6 ± 0.1	1.04

EDOT-GFF and EDOT-GFFD could both be best fitted using a flexible elliptical cylinder model at high and low pH. Chi squared (χ^2^) values, that quantify the goodness of fit, markedly improved at low pH suggesting a more homogeneous and ordered system (Table 1 and Table S1). It was interesting to note that while the inclusion of aspartic acid to the peptide sequence maintained an elliptical cross section (EDOT-GFFD), its absence (EDOT-GFF) indicated a possible circular to elliptical cross section transition with decreasing pH (Table S1). Such a change has been previously observed in diphenylalanine-containing gelators.^33^ It should be noted that an acceptable fitting was found with a flexible cylinder model in all post-GdL cases. A hollow cylinder model was preferred in the case of EDOT-FFF as the peak at higher Q was indicative of a hollow system as seen in previous work.^33^ The flexible cylinder model fitted EDOT-FFF better at lower Q but may well be overfitted and artificially biased due to larger aggregates. It is interesting to note, however, the long Kuhn lengths (Table S1) observed with a flexible cylinder compared to the other gelators suggesting a more rigid and linear structure in EDOT-FFF.

**Figure 5:**
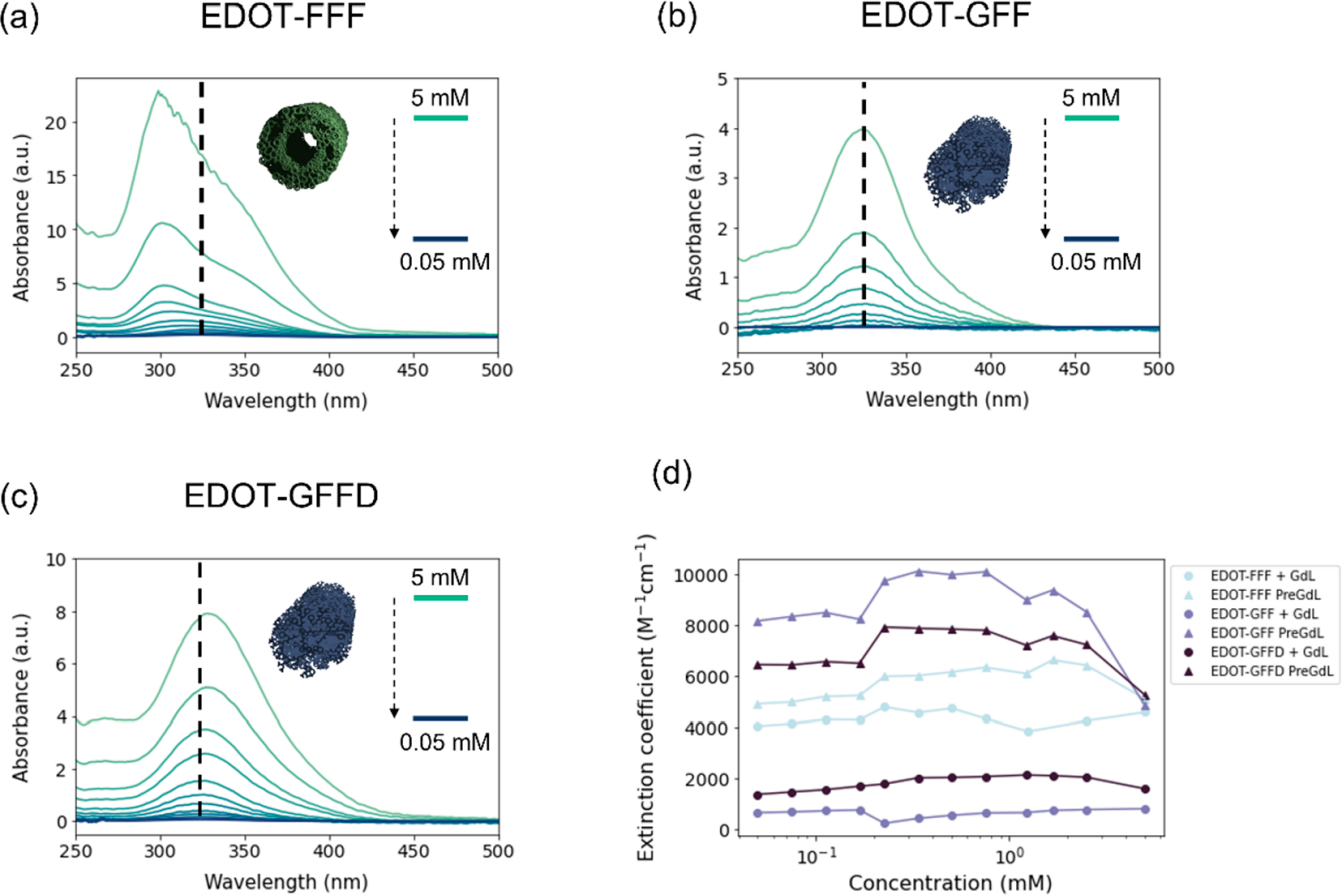
Normalized UV–visible (UV–vis) spectroscopy dilution data for (a) EDOT-FFF, (b) EDOT-GFF, and (c) EDOT-GFFD; 12 measurements were taken across the concentration range. Dashed lines illustrate where the peaks tend toward as concentration is decreased. (d) Extinction coefficients with dilution for the three gelators at high pH and low pH in water (i.e., before and after gelation).

Comparing the EDOT-peptide gelators, it appears in the absence of a flexible glycine linker for EDOT-FFF, the system adopts a hollow cylinder morphology at high pH (Figure 4d) that is preserved down to low pH, possibly due to the directionality, enforced by potential π–π interactions of the aromatic residues (debate exists around the prevalence of actual face-to-face “pi-stacking” as a dominant driving force)^34^ coupled with a lack of flexibility. This is in concord with the kinetic AFM measurements (Figure S5) where EDOT-FFF appears to move from loose to tightly bundled fibers. Such aligned, face-to-face arrangement might be a result of the enforced directional aromatic interactions possibly from the H-like aggregation^35^ suggested by a blue shift and broadening in the UV–vis dilutions (Figure 5a) that leans energetically toward a hollow structure.^36^ In this data we attribute the main absorption peaks at around 350 nm to the EDOT moiety whose conjugation is extended when coupled to an oxalyl group.^27^

Conversely, EDOT-GFF and EDOT-GFFD gels assemble through a movement from circular-to-elliptical or elliptical-to-elliptical cylinders, respectively, according to SANS. In both cases, the SANS data contain substantially greater noise at high pH, particularly EDOT-GFF that had large uncertainties in fitting (Figure S4 and Table S1). Since fibers were not observed pre-GdL by AFM (Figure S5), it is possible these were more heterogeneous systems than EDOT-FFF that contained a mixture of aggregates such as cylinders and even spheres or short worm-like micelles at concentrations such that clear fitting was not possible. If adopting a spherical-to-worm-like micelle transition,^22^ it is hypothesized these systems might inhabit a kinetic space between the two that subsequently tend to elliptical fibers with decreasing pH. The preference for the more hydrophilic EDOT-GFFD and more flexible EDOT-GFF to exhibit elliptical structures at low pH was accompanied by peak broadening at high concentration (Figure 5b,c) and decreasing extinction coefficients with dilution (Figure 5d—post-GdL data) which was also taken to imply H-like aggregation.^34^ It should be noted that we do not attempt to speculate at this point about the exact nature of the supramolecular arrangement (e.g., β sheet vs α helix). Techniques such as cyclic dichroism (CD) have been used to this effect in the past but have often yielded different results for the same compound and therefore can be unreliable in ascertaining such information.^22^

Examination of the extinction coefficient (ε) with decreasing concentration from 5 to 0.05 mM (Figure 5d) was determined using UV–vis spectroscopy. It was reasoned changes in ε could reveal transformations in assembly with dilution that might afford better understanding of the energetic landscape. Initially, the gelators were profiled in DMSO (Figure S6) in which they were assumed to be monomeric, providing a marker for complete dissolution of the compounds in terms of ε. This assumption is derived from literature precedent of similar peptides in which DMSO is commonly used as the solvent when a “solvent/antisolvent” trigger is employed.^37,38^ When comparing profiles before and after gelation, it was found for both EDOT-GFF and EDOT-GFFD that no convergence was seen, indicating that dilution does not revert the structures back to their pre-gelation state, and the aggregated state of the molecules is the most stable. This is in concord with the AFM data taken throughout assembly (Figure S5), where a movement from random or spherical aggregates to bundled fibers was observed representing two distinct phases. Conversely, EDOT-FFF demonstrated some convergence between the pre- and post-gelation samples with dilution suggesting that a low concentration might afford similar structures at both high and low pH. AFM images taken throughout the assembly indeed reveal fiber-like structures for EDOT-FFF (Figure S5) at high pH indicating dilution transforms the structures possibly from a bundled fiber, that is more exaggerated at low pH, to separate fiber morphology.^22^ These data highlight how care must be taken in concluding to what extent microscopy, often taken of diluted samples, is representative of the bulk morphology.

### Thermodynamics of Dissociation

**Figure 6:**
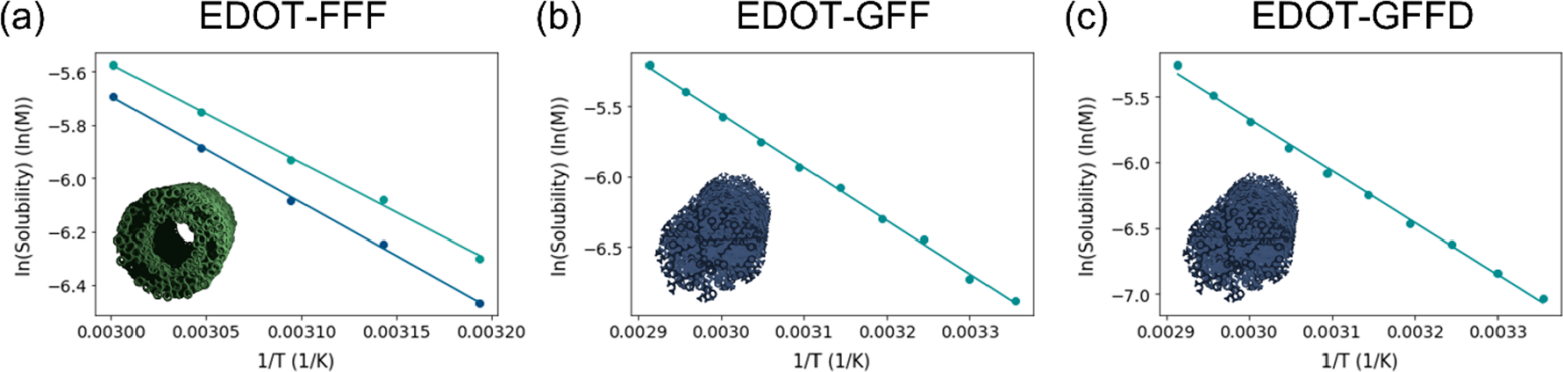
Van’t Hoff plots of (a) EDOT-FFF before (green) and after (blue) GdL addition, (b) EDOT-GFF after GdL addition, and (c) EDOT-GFFD after GdL addition. Circular points are the data and line plots are linear best fit lines. No linear plots were obtained pre-GdL (high pH) for EDOT-GFF and EDOT-GFFD.

To further understand the driving forces behind the respective assemblies, we wanted to probe the thermodynamics inherent to the systems, as this was believed to be key to understanding the self-assembly and thus any charge transport implications. Solubility profiling with temperature has been previously shown in organic solvents to grant estimations for thermodynamic values such as dissociation enthalpy and entropy but to the best of our knowledge has not been applied to aqueous systems.^39^ To estimate these values, we employed the following Van’t Hoff equation:
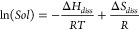
1where *Sol* is the solubility, Δ*H*_*diss*_ and Δ*S*_*diss*_ are the enthalpy and entropy of dissolution, respectively, *R* is the molar gas constant, and *T* is the temperature. To employ ^1^H NMR in this way, we assume that the system behaves cooperatively, i.e., that molecules are effectively either free monomers or part of the larger self-assembled structure without forming smaller oligomer intermediates. Second, we assume that all large self-assembled structures will be NMR silent, since the size of the structures will result in slow molecular tumbling and hence long T1 values. By undertaking ^1^H NMR measurements across a temperature range, eq 1 allows the extraction of thermodynamic constant estimates by plotting the natural log of solubility against 1/*T* (Figure 6). Solubility values, or concentrations of the compounds that are NMR visible, are extracted from the spectra by comparison of the phenyl protons with concentration of a known internal standard (Figure S7). The experiment was conducted both before and after GdL addition to probe the thermodynamics at both high and low pH, respectively.

Surprisingly, pre-GdL solutions of EDOT-GFFD and EDOT-GFF appeared monomeric or short-oligomeric in solution by ^1^H NMR despite SANS data suggesting the presence of a structure (Figure 4b,c). This might result from an equilibrium between a smaller population of assembled structures and a larger population of mono/oligomeric, ^1^H NMR-visible species which could explain why fibers were not obvious by AFM (Figure S5). Conversely, EDOT-FFF at high pH was not monomeric, showing broad peaks (Figures 6a and S7a) which suggests the presence of large, aggregated structures again, in agreement with AFM (Figure S5).

At low pH, all three gelators gave linear ln(*Sol*) versus 1/*T* plots (Figure 6a–c) from which thermodynamic constant estimates were extracted (Table 2). It was seen that EDOT-FFF shows the highest enthalpy of dissociation that suggests the intermolecular forces in the assembly are the strongest of the gelators. It also shows the greatest entropy of dissociation, indicating the highest degree of order when assembled, a conclusion in agreement with the long Kuhn length from the SANS data (Table S1) and fiber stability to high pH observed in SANS, UV–vis, and AFM. In comparison, EDOT-GFF and EDOT-GFFD both gave significantly lower enthalpies of dissociation than EDOT-FFF, indicating weaker intermolecular interactions. Smaller differences were seen in the entropy values with EDOT-FFF > EDOT-GFF > EDOT-GFFD. It seems although addition of aspartic acid might yield straighter and thinner fibers, EDOT-GFF still retained a higher degree of order. It is also interesting to note that despite having lower energetic penalties of dissociation than EDOT-FFF, EDOT-GFF was able to exhibit the highest storage modulus (Figure 2). This suggests the overall mechanical strength of such gels is not purely a function of its intermolecular interaction and structural order but also of other relations. One possible origin could be fiber solvation and degree of fiber bundling, more of which is suggested to occur in EDOT-GFF by the spread into larger radii found in its AFM histogram (Figure 3b).

**Table 2:** Energetic Parameters Estimated by ^1^H NMR Van’t Hoff Analysis^a^

Compound	Δ*H*_*diss*_, kJ mol^–1^	Δ*S*_*diss*_, J mol^–1^ K^–1^
EDOT-FFF	38.34 ± 1.13	52.87 ± 6.66
EDOT-GFF	31.16 ± 0.35	48.02 ± 1.08
EDOT-GFFD	30.34 ± 0.82	44.23 ± 2.47

a Δ*H*_*diss*_ and Δ*S*_*diss*_ refer to the enthalpy and entropy of dissociation, respectively.

Interestingly, assessing the appropriate temperature range in which the compounds remain as gels by rheology (Figure S8) revealed surprising phenomena. While EDOT-FFF lost structural integrity beyond 60 °C, EDOT-GFF remained stable from 30 to 70 °C and EDOT-GFFD actually increased in storage modulus continuously to 80 °C despite as much as 50% of the gelator being ^1^H NMR visible and, we assume, free in solution at this temperature. This unexpected result suggests increased temperature encourages structural rearrangements that further strengthen the gel state. Indeed, the modulus increases even faster upon cooling with a total increase in over 2 orders of magnitude over the whole cycle (Figure S8d). Similar studies have stipulated this may be the result of lengthening of coiled fibers during dehydration with increasing temperature.^40^ It is interesting to note that this phenomenon was only seen in EDOT-GFFD, suggesting a distinctly different form of assembly. One possibility is, when considering this gelator showed the lowest entropy to be released upon dissolution (Table 2), that this system might exist in a kinetically stable state and transitions to a more thermodynamically stable configuration exhibiting a greater modulus upon heating.

Previous examples using these peptide sequences can be considered for reference. Examples using FFF have been found to produce fibers when capped with Fmoc or boronic acid on the nanometer diameter scale.^41,42^ However, in general, no in depth study of the specific fiber morphology such as that of neutron scattering is present, and as such, direct comparisons are limited. Further, these studies utilize different assembly triggers, adding to the challenge of their use as a cross reference. More broadly, assembly from ethanol evaporation into ordered, crystalline nanospheres was observed when FFF is capped with a Boc group possibly hinting at the sequences’ preference for ordered, tightly bound assembly as we suggest here.^43^ Examples of fibers from GFF and GFFD containing molecules in the literature also exist though variety in proposed stacking (herringbone vs face-to-face) can be seen with variation in capping moiety.^44−46^ Again, different assembly triggers were chosen, and elucidation of the specific fiber morphology is unfortunately generally absent again preventing direct comparison with our systems.

### Molecular Dynamics Simulations

To have greater understanding as to the specific intermolecular interactions that guide assembly of the gelators, we turned to molecular dynamics (MD) simulations (for approach and methodology refer to Supporting Information and Figure S9, Tables S2 and S3). Systems of one, two, and 20 EDOT-GFF, EDOT-GFFD, EDOT-FFF, EDOT-FF, and EDOT-FFD monomers in an aqueous environment were simulated to determine the mode/geometric features of association, the residues of the EDOT-peptides responsible for association, and how these play a role in the self-assembly of the observed supramolecular morphologies.

**Figure 7:**
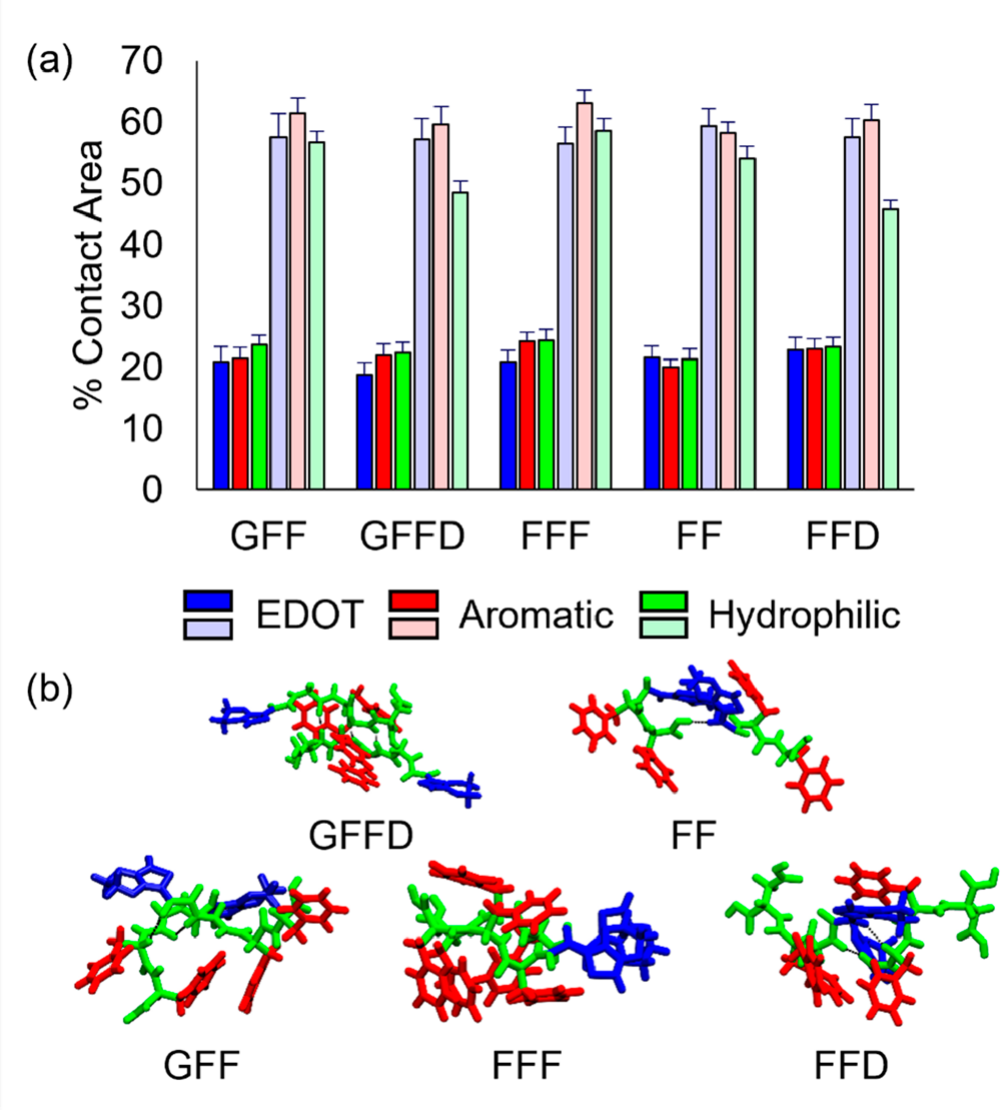
(a) Average contact surface area for EDOT, aromatic, and hydrophilic backbone residues from molecular dynamics (MD) simulations of dimer (shaded) and oligomer (transparent) EDOT-GFF, EDOT-GFFD, EDOT-FFF, EDOT-FF, and EDOT-FFD assemblies compared to the solvent accessible surface area of a single EDOT peptide monomer. (b) Representative structures from MD simulations of interacting EDOT-GFF, EDOT-GFFD, EDOT-FFF, EDOT-FF, and EDOT-FFD peptide dimers, indicating preferred interactions leading to their self-assembly behavior. Solvent molecules and periodic unit cells are not displayed for the sake of clarity.

To monitor the evolution of the self-assembling peptide systems, and to reveal the specific interactions that drive the peptides’ behavior, we discriminate between peptide regions that remain solvent exposed and those that intermolecularly associate upon assembly. This is achieved by monitoring the solvent accessible surface area (SASA) of a single EDOT-peptide in solution and comparing to the SASA of interacting peptides in the systems with two (dimer) and 20 monomers (oligomer). The average surface area in contact (CA) for each peptide-monomer in the dimer/oligomer assembly is used to quantify the degree of association and is defined as
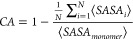
2where ⟨...⟩ denotes time-average and N is the number of peptides in the system (2 or 20). We distinguish the CA contributions of the EDOT moiety, aromatic side-chain groups, and the hydrophilic peptide backbone to compare the preferred interactions between the different EDOT-peptides and relate this to their observed self-assembly behavior. Figure 7a plots the average contact surface area for each of the distinct residues in the dimer and oligomer as a percentage of the solvent accessible surface area (SASA) of that residue in a single EDOT-peptide monomer.

Figure 7b presents representative structures for EDOT-peptide monomer interactions colored for the distinct residues plotted in Figure 7a. For the simulations containing two EDOT-peptide monomers, EDOT-GFF, EDOT-GFFD, EDOT-FFF, EDOT-FF, and EDOT-FFD monomers were in contact with each other on average for 46%, 67%, 72%, 38%, and 41%, respectively, in the final 100 ns of simulation. This demonstrates the stronger association of the EDOT-peptides that self-assemble into fibers (EDOT-GFF, EDOT-GFFD, and EDOT-FFF). Figure 7a shows for the fiber-forming EDOT-peptides, there is a stronger tendency for the monomer interactions to involve the aromatic and hydrophilic residues than the EDOT moiety, while the opposite is true for EDOT-FF, and there is no clear preference for EDOT-FFD. Representative structures in Figure 7b for EDOT-peptide monomer interactions demonstrate π–π interactions between phenylalanine rings in EDOT-GFF, EDOT-GFFD and EDOT-FFF. In EDOT-GFF and EDOT-GFFD, the flexibility of the glycine linker promotes hydrogen bonding between peptide backbones and structural rearrangement to afford complementary π–π interactions. Conversely for EDOT-FFF, the lack of glycine reduces flexibility and results in predominantly π–π interactions, with hydrogen bonding between the peptide backbones less accessible. As EDOT-peptides engage in π–π interactions, the backbone of the peptide becomes less accessible to the solvent, so naturally the relative intra- and interpeptide contact area increases. As the interacting EDOT-FFF backbones appear to be less accessible to the solvent, the backbone hydrogen bonding is present on average for only 47% of the time in contact, compared to 52% and 59% for EDOT-GFF and EDOT-GFFD, respectively. In MD simulations of larger oligomeric assemblies of 20 EDOT-peptide monomers, we observe π–π interactions as the main driving self-assembly mechanism for all EDOT-peptides. Hydrogen bonding between hydrophilic moieties also contribute to EDOT-peptide association, particularly for EDOT-GFF where the flexibility of the glycine linker promotes oligomerization. For EDOT-GFFD however, and EDOT-FFD, the l-aspartic acid terminal favorably hydrogen bonds with water, remaining accessible to the solvent. This is clear in Figure 7a, with a marked reduction in the contact area of the peptide backbone compared to the EDOT and aromatic residues for EDOT-GFFD and EDOT-FFD.

The simulated self-assembled structures of 20 EDOT-peptide monomers clearly demonstrate the tendency to arrange with externally facing (water-exposed) hydrophilic residues and internally shielded π–π interactions (Figure S9). For example, the dominant side chain π–π interactions that prefer to internalize within the EDOT-FFF assembly are accommodated by parallel peptide arrangements that intermolecularly hydrogen bond across peptide–peptide backbones. This is evident in Figure S9 when visualizing the EDOT-FFF monomer secondary structure elements. In contrast, the flexible glycine linkers of EDOT-GFF and EDOT-GFFD allow peptide monomer backbones to form more interconnected hydrogen bond networks across the oligomeric assemblies. This enhances the β-like conformational characteristics of their secondary structure compared to EDOT-FFF, with emerging β sheet-like alignments for EDOT-GFF and identifiable small β sheets for EDOT-GFFD. The hydrophilic peptide backbone also interacts with the solvent in the case of EDOT-GFFD. From these basic observations, it can be suggested that the enhanced aromatic character of EDOT-FFF limits hydrogen bonding between larger aggregates, and this may contribute to the formation of hollow macromolecular structures rather than elliptical.

It is worth noting that spontaneous peptide self-assembly on the experimental time scale is currently out of reach for all-atom MD simulations;^47^ therefore prototypical model systems have been employed to provide insights into the intermolecular interactions driving the self-assembly process. Similar MD simulations have previously been successful in rationalizing different peptide self-assembly mechanisms in corroboration with experimentally observed morphologies.^48,49^

Reflecting on this, it is possible that a more hydrophobic and aromatic molecular design like EDOT-FFF enforces more solvent shielding, encouraging intermolecular association with greater enthalpy and entropy released upon dissolution than the other gelators (Table 2). The stronger preference to shield from the solvent may have resulted in a kinetically trapped state—the fibrous gel. Further evidence for this is seen when application of heat quickly destroys such ordered architectures, indicating the more thermodynamically stable state is not a fiber gel assembly (Figure S8). Inclusion of glycine in EDOT-GFF and EDOT-GFFD, however, seems to prefer more open, hydrogen bonded structures, more loosely bound with less entropy released upon dissolution (Table 2): perhaps a result of a greater number of possible conformations afforded by a flexible linker. These structures seem to survive heating (Figure S8) and even increase in modulus for EDOT-GFFD suggesting rearrangements into more mechanically robust assembled conformations. It is worth noting these differences in moduli response to heating may demonstrate that the two elliptical fiber examples, EDOT-GFF and EDOT-GFFD, differ in assembly mechanism and molecular arrangement, meaning three distinct assembly modes might actually be present in this study.

### Charge Transport

**Figure 8:**
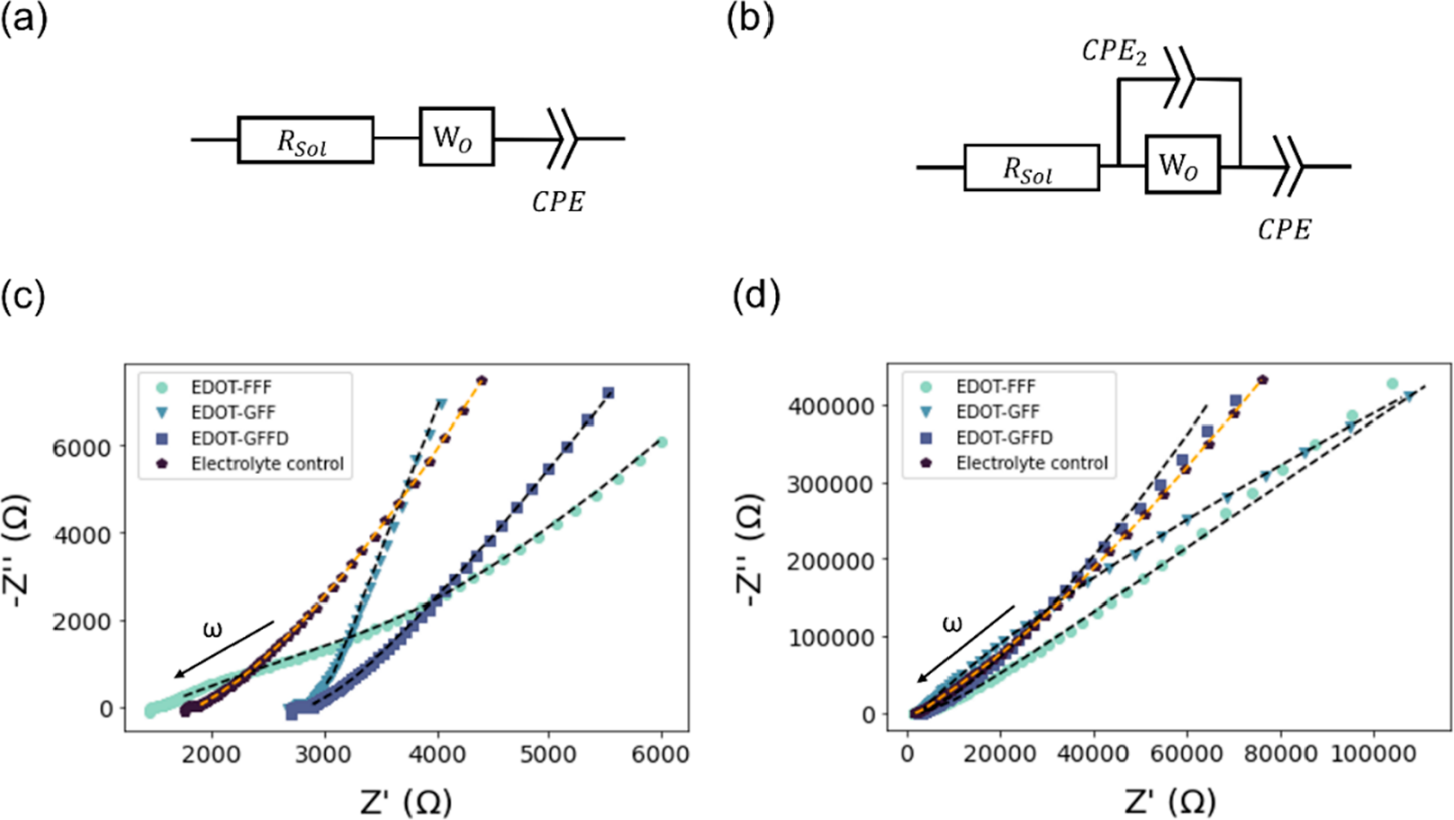
(a) A simple equivalent circuit describing a gel (or electrolyte) in contact with an inert glassy carbon electrode, ignoring the high frequency loop seen in the impedance spectra. The constant phase element (CPE) represents the electrolyte double layer at the electrode interface. The finite-length Warburg element^39^*W*_*O*_ represents the diffusive accumulation and dissipation of charge at the interfaces and *R*_*sol*_ is the bulk solution resistance. (b) A modification of the gel-electrode equivalent circuit in (a) to include dispersive accumulation/dissipation of charge at the interfacial region or dispersive transport there. (c) Impedance data from the high-to-mid-frequency range; (d) full impedance spectra from 10^6^ to 0.1 Hz except for the EDOT-GFF where the full range is shown in Figure S10. Equivalent circuit fits are shown by the black dashed lines, with the fit for the electrolyte control shown as an orange dashed line. EDOT-FFF and EDOT-GFF are fit with (b) while EDOT-GFFD and the electrolyte control are fit with (a); the fitting parameters are given in Table S4.

Following the characterization and simulation of the structures and assembly pathways, we assessed charge transport through the gels. Conductivity measurements of the hydrated gels using a four-point probe (4PP) showed EDOT-FFF exhibited nearly twice the conductivity of EDOT-GFF and EDOT-GFFD (Table 3). Measurements of corresponding samples in the dry state yielded 2–3 orders of magnitude lower conductivities (10^–5^–10^–6^ S/m, data not shown). Since we used only single EDOT units in the synthesis, this observation combined with the absence of a major DC conduction contribution to the impedance spectra (Figure 8c,d)^50^ of the hydrated gel samples indicates charge transport in the gel is primarily ionic with no Faradaic process at the electrodes, and that the contribution from conducting holes through the fibers themselves is negligible. As a comparison, the conductivity of a GFF sequence with an Fmoc capping group rather than EDOT was tested. It was observed that this gelator gave an average conductivity of 0.057 ± 0.031 S/m, a value between those of EDOT-FFF and EDOT-GFF/EDOT-GFFD. However, since this system was not structurally profiled, the conclusion drawn is that EDOT capping groups, in and of themselves, do not appear to drastically enhance conductivity over other aromatic termini and that such charge transport values might pertain to the field more broadly.

**Table 3:** Gel Conductivity Estimates from the Four-Point Probe and the Real Part of the High Frequency (1 MHz) Electrochemical Impedance

Compound	Conductivity by four-point probe (*c*), S/m	Conductivity by impedance spectroscopy (*σ*_*EIS*_), S/m
EDOT-FFF	0.0812 ± 0.022	0.0248 ± 0.006
EDOT-GFF	0.0379 ± 0.007	0.0137 ± 0.001
EDOT-GFFD	0.0408 ± 0.006	0.0137 ± 0.002
Electrolyte	n/a^a^	0.014 ± 0.002

a Four-point probe measurements not conducted on the electrolyte itself since a sample thickness could not be accurately ascertained given, for instance, the convex meniscus.

Nyquist plots showing the electrochemical impedance at different frequencies of the gels and control electrolyte sample are shown in Figure 8 and allow further insight into the conduction mechanisms. It is reasonable to assign the intercept of the high frequency impedance with the *x*-axis (real part) on the Nyquist plot (Figure 8c,d) to the solution resistance, inversely proportional to the bulk conductivity of the solution. The relative differences between the gels are consistent with the four-point-probe measurements, where the EDOT-FFF gel showed about half the resistance of the EDOT-GFF and EDOT-GFFD gels. The absolute differences between the DC 4PP and the high frequency EIS conductivity values are within an order of magnitude and are related to the differences between the techniques and system geometry. Interestingly the electrolyte control sample showed on average a similar series resistance to the EDOT-GFF and EDOT-GFFD samples; in contrast, the EDOT-FFF sample showed a lower resistance than the electrolyte control, consistent with a higher ionic mobility and/or ion activity (effective concentration) in this gel structure. It should be noted, if tempted to ascribe this increase relative to the electrolyte to the gelator itself diffusing, the Van’t Hoff NMR analysis showed EDOT-FFF to be the least present in solution once assembled (Table 2 and Figure S7). For reference, conductivity values typically reported in biomaterials designed for charge transport through incorporation of conducting polymers range from 10^–4^–10^0^ S/m.^51^

We note that the Nyquist plots for all gels and the electrolyte control showed a small loop at high frequency with a width of approximately 200 Ω. This feature is often attributed to the capacitance of the electrical double layer at electrolyte interfaces.^52^ However, we were unable to obtain repeatable and reliable measurements of this region, so to avoid overinterpretation, we have excluded this feature from our models. A reasonable fit to the electrolyte control sample data, excluding the high frequency loop, can be achieved using the circuit model shown in Figure 8a. In this model the inert, non-Faradaic, glassy carbon electrode interface is described by a constant phase element (*CPE*) in series with an “open” Warburg element (*W*_*O*_)^53^ describing the diffusive accumulation and dissipation of ionic charge at the interface and a series resistor (*R*_*Sol*_) describing ionic transport through the bulk. This model also described the behavior of the EDOT-GFFD gel well. In general, an additional constant phase element (*CPE*_*2*_ - full circuit shown in Figure 8b) in parallel with the Warburg element was introduced to better describe the behavior of the EDOT-FFF and EDOT-GFF gels (Figure 8c). This could be consistent with adsorption and desorption of ionic charge within an extended interfacial region near the electrode interface leading to the signatures for dispersive processes that we observe in the impedance spectra.

An alternative explanation for the dispersion in RC relaxation times associated with the *CPE*_*2*_ element used to fit the EDOT-FFF and EDOT-GFF data could be related to these gels exhibiting an inhomogeneous fractal structure leading to frequency dependent transport through the gel network near the electrodes.^54^

**Figure 9:**
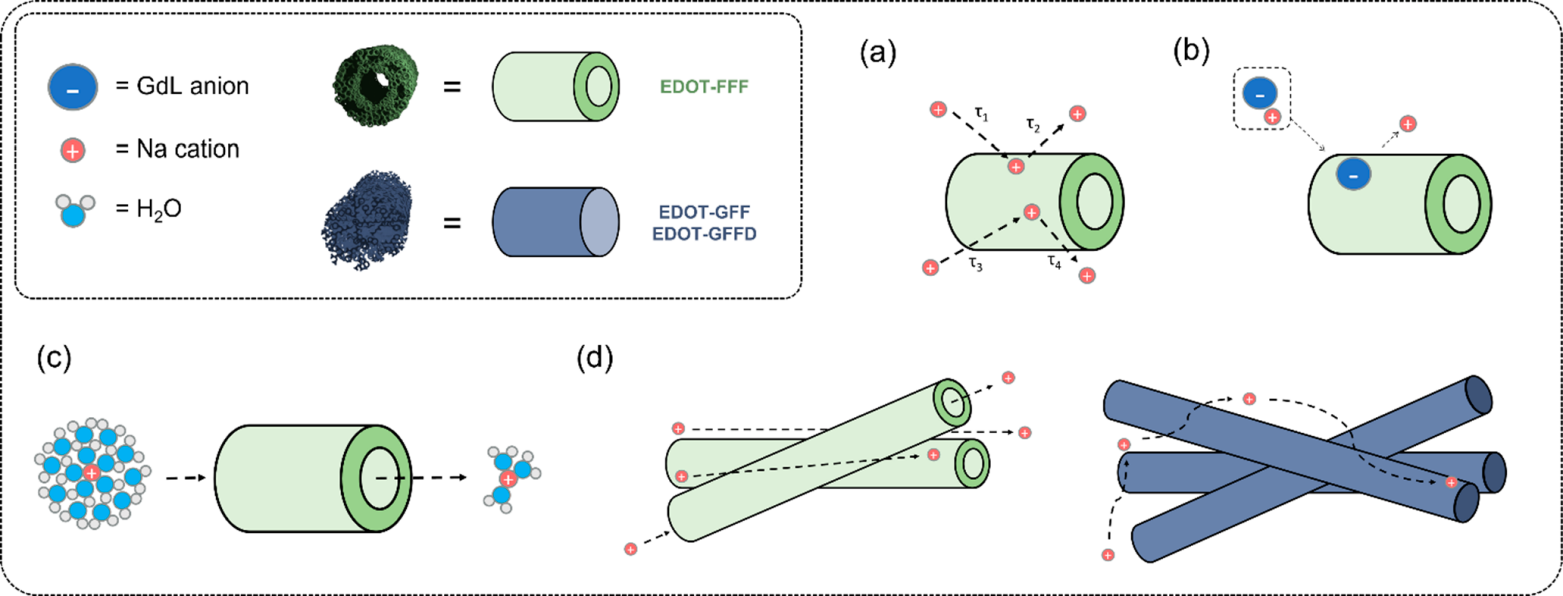
Schematic representation of the proposed mechanistic origin of the conductivity differences observed by experiment. (a) Illustration of different rates of absorption–desorption on the fiber surfaces; (b) separation of paired ions via binding to the fiber surface disrupting their net neutrality; (c) disruption of the solvation shell of the ions; and (d) differences in tortuosity and constrictivity between hollow and solid fibers that may also contribute to differences in conductivity. Here the blue tubes represent the elliptical cylinder systems of EDOT-GFF and EDOT-GFFD. Note that although illustrated here primarily with the hollow tube of EDOT-FFF, the mechanisms are speculated to be occurring in all gelators, particularly the fiber surface-based phenomena of (a) and (b).

We now briefly reflect on the possible origins (Figure 9) of the differences observed in the electrical measurements since we assume all samples have the same background concentration of ions. The high conductivity of the EDOT-FFF gel relative to both the other two gels and the electrolyte control might be explained by one of, or some combination of, the following:

1. Differences in the rates and scale of surface adsorption and release of conducting ions on the gel fiber surfaces. Repeated capture and release of ions on the network’s surface could reduce the effective mobility of ions through the system—also resulting in a distribution of time constants for capacitive and transport effects in the gel (Figure 9a). If the EDOT-GFF and EDOT-GFFD gels have higher propensity toward these processes, then it could explain their lower conductivity. We note that this explanation in isolation is not consistent with the control electrolyte showing a similar conductivity to these gels.
2. A higher concentration of active ions relative to the other samples. This could be plausible if ion pairing occurs in the electrolyte (which would reduce conductivity—Figure 9b). Preferential adsorption of one of the electrolyte ion species onto the gel fiber surface could conceivably result in a reduction in ion pair concentration, releasing unpaired ions for conduction (i.e., increase the electrolyte activity). The structural data indicates the EDOT-FFF gel fibers are hollow and likely to have a higher surface area per unit volume than the other samples which could be consistent with this hypothesis.
3. A higher mobility of the ions. The structure of the EDOT-FFF gel may in some way disrupt the solvation shells around electrolyte ions in this gel, increasing their mobility by reducing the solvation shell radius (Figure 9c). Conceivably, this could occur within the hollow EDOT-FFF fibers.
4. A reduction in the tortuosity and constrictivity of the gel structure. The pathways for ion transport could be more open with fewer circuitous routes and dead ends in the EDOT-FFF gel relative to the other two gels acting as an artificial “ion channel”, resulting in higher ionic conductivities (Figure 9d). Again, we note that this explanation in isolation is not consistent with the control electrolyte showing a lower conductivity.

Further study is required to confidently ascertain the origins of the observed differences in electrical properties, but our results indicate that small differences in the molecular design of the gels can significantly influence their performance. We have shown that the morphology of assembly can be dictated through chemical and thermodynamic design, in particular with triphenylalanine adopting hollow cylinders. If enhanced ionic transport were to be mediated by these hollow fibers, interesting bioengineering possibilities would arise since ionic motion is a ubiquitous form of cellular communication often relying on ion channels in conventional biological tissue.

In conclusion, the EDOT moiety has been successfully incorporated into a self-assembled hydrogel system with peptide sequences spanning a range in hydrophobicity. An array of characterization techniques highlighted the impact small changes in peptide sequence have on the thermodynamics of dissociation in solution that in turn inform the assembly pathway. We observed that the selection of either hollow or elliptical cylinders could be dictated by molecular design. We further propose such differences arise from a combination of more hydrophobicity in the peptide sequence, such as three phenylalanines, causing greater energy cost for dissociation coupled with possible directional bonding from conjugated residues and structural rigidity (as informed by a glycine spacer). From our results it appears use of more rigid and hydrophobic molecular design such as EDOT-FFF can tend assembly toward more defined structures such as hollow nanotubes (Figure 4) with greater enthalpy and entropy of dissolution. These maintain their morphology across the pH range of the trigger with the increasing acidity likely serving to encourage more bundling and fiber association, allowing gel formation. Further, these structures are likely more tightly associated and ordered as indicated by the enthalpy and entropy of dissociation estimates in Table 2 as well as potentially stacked face-to-face as H-like aggregates (Figure 5). Indeed, MD simulations suggest that EDOT-FFF prefers π–π interactions that shield the molecular backbone from solvent interfacing and reducing hydrogen bonding. This rigidity, order and large entropy release upon dissolution may also explain the mechanical failure upon heating in contrast to the stability and even thermal strengthening seen in its less rigid and less hydrophobic counterparts (Figure S8).

Increasing flexibility (glycine inclusion) and hydrophilicity (aspartic acid inclusion) appears to yield solid elliptical fibers with potentially less order, as suggested by the entropy change (Table 2) and no dominant aggregation character, as indicated by the lack of λ_max_ shift with dilution (Figure 5). MD simulations further suggested these compounds relied less upon π–π interactions, giving more solvent-exposed molecular backbones, greater hydrogen bonding, and possible β-sheet arrangement or β-sheet like alignments (Figure S9). We speculate that while assembly into fiber-based gels can be broadly achieved in peptide derivatives through desolvation via a pH trigger, the specifics of the ordering within the fibers itself are highly amino-acid sequence dependent. With this said, great care must be taken in generalizing from such data as design rules are notoriously difficult to establish in low molecular weight gelators.^29^ Nonetheless, the pathways of assembly investigated in this study have been shown to dictate the mechanical and morphological properties, which in turn may influence changes in charge transport. Indeed, a small alteration in the molecular design led to a doubling in the electrical conductivity of the solid ionic medium. We speculate that this could originate from the hollow nature, or lack therein, of the fiber structures influencing the electrical properties of the gel. A number of possible mechanisms behind this phenomenon have been proposed including surface absorption–desorption effects, tortuosity of charge diffusion pathways, ion pair separation, and solvation shell disruption. Charge transport enhancement is generally anticipated to elevate the integration with, and facilitation of communication between, biological tissues and is indeed strongly linked to the differentiation and proliferation of appropriate cell types (neuron, cardiac etc.).^1−5^ Systems constructed from common building blocks of the biological milieu such as amino acids are anticipated to be highly cytocompatible and nonimmunogenic allowing for seamless integration with biological tissue. For the LMWG material field that can already mimic extracellular tissue morphology and mechanical properties, and offer extensive “self-healing” properties, the understanding of how to program enhanced ionic charge transport through elucidated molecular design is anticipated to progress the field toward the complexity demanded of it.

## Experimental Section

### General Remarks

All solvents were purchased from Fischer scientific or VWR. All reagents were purchased from Sigma-Aldrich unless stated otherwise below. 2-Chlorotrityl chloride resin (1.0–1.2 mol equiv/g 200–400 mesh) was purchased from Chem-Impex International Inc. Fmoc Asp(OtBu) was purchased from Fluorochem. 3,4-Ethylenedioxythiophene (EDOT) was purchased from Alfa Aesar. 1-[Bis(dimethylamino)methylene]-1*H*-1,2,3-triazolo[4,5-*b*]pyridinium 3-oxid hexafluorophosphate (HATU) and *O*-(1*H*-6-chlorobenzotriazole-1-yl)-1,1,3,3-tetramethyluronium hexafluorophosphate (HCTU) coupling reagent were purchased from AGTC bioproducts. Deuterated chloroform and dimethyl sulfoxide (DMSO) were supplied by MagniSolv. Deuterated water, water, acetonitrile, sodium hydroxide, tetrahydrofuran (THF), *n*-hexane, and magnesium sulfate were all purchased from VWR. Ethyl acetate and hydrochloric acid were purchased from Fischer scientific. *N*-Methyl-2-pyrollidone was purchased from Thermo scientific.

Column chromatography was undertaken using silica gel (40–63 μm) and visualized on a thin film chromatograph using UV irradiation (254 nm wavelength). ^1^H and ^13^C nuclear magnetic resonance (NMR) measurements were made using a JEOL autosampler NMR spectrometer at 400 and 75 MHz respectively and conducted at 298 K unless otherwise stated. Chemical shifts (δ) were referenced to the residual peak of the solvent (CDCl_3_ = 7.26 ppm, DMSO-*d*_6_ = 2.50 ppm or D_2_O = 4.79 ppm) unless stated otherwise. Proton (^1^H) NMR data are reported as chemical shifts with the following multiplicity notation: s = singlet; d = doublet; t = triplet; q = quartet; m = multiplet; br = broad; td = triplet of doublets. This is followed by the proton position and then the coupling constants (*J*) in Hertz if applicable. High-resolution mass spectrometry (HRMS) was undertaken using an Agilent 6546 liquid chromatography/quadrupole time-of-flight (LC/Q-TOF) mass spectrometer. This was run in a 50–70% gradient of acetonitrile in water with 0.1% formic acid over 6 min using an EclipsePlusC18 RRHD 1.8 μm, 2.1 × 50 mm column at a flow rate of 0.5 mL/min. Mass calculations were done using the Agilent MassHunter qualitative analysis 10.0 software.

### Assembly Protocol

In a typical experiment, the EDOT-peptide was dissolved in 1.5 equiv of NaOH in MQ water (1 M stock). Sonication was undertaken for 10 min to ensure dispersion of the compound in solution. To this was added 2 equiv of glucono-δ-lactone (GdL) (predissolved in MQ at ∼0.5 M and immediately added) giving the final concentration of gelator as 10 mM. At this point the gelation process had begun, if experiments were undertaken in the gelled state a minimum of 12 h was required before measurements were taken.

### Atomic Force Microscopy

Measurements were performed on a 5500 Agilent AFM instrument using an AC240TS-R3 cantilever from Oxford Instruments (*f* = 70 kHz, *k* = 2 N/m, no tip coating) with AC tapping mode in air. Typically, substrates were prepared on Si by diluting down to 0.1 mM then applying 5–10 μL to the surface for 10–20 min following which a Kimwipe was used to wick off remaining liquid. Samples were imaged within 48 h of preparation. Images were processed using Gwyddion (64 bit) software including the use of flattening and polynomial row alignment functions. When obtaining height profile histograms, a minimum of 50 measurements were made for each gelator type.

### UV–Visible Absorbance Spectroscopy

UV–vis absorbance was measured using a Nanodrop 2000c spectrometer. Samples were measured in quartz and sometimes optical glass cuvettes with path lengths ranging from 0.1 to 2 mm.

### Extinction Coefficient

The (molar) extinction coefficient (M^–1^ cm^–1^), ε, values were calculated by using the following equation:
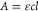
where *A* is the absorption, *c* is the molar concentration (M), and *l* is the path length (cm).

### Rheology

Rheology measurements were taken using an Anton Paar MCR 302 Modular Compact Rheometer. Samples were measured using a 25 mm plate with parallel plate geometry. Time-resolved sweeps were conducted at a constant frequency = 1 Hz, strain of 0.1% and at steady state with max equilibration time of 200 s. A data point was collected every minute for 12 h. To justify the frequency and strain values used, a frequency sweep (*f* = log sweep 100–0.01 Hz, strain = 0.1%, steady state with max equilibration time = 200 s, 10 data points per decade—Figure S3a) and strain sweep (*f* = 1 Hz, strain log sweep 0.01–100%, steady state with equilibration time = 20 s—Figure S3b) were conducted. It was observed that the gels displayed stable storage and loss modulus behavior in the values chosen for the time sweep.

### pH Profiling

A Mettler Toledo FiveEasy plus pH meter was used. The equipment was calibrated using a three-point calibration to 4, 7, and 10 pH standards before use. The probe tip was rinsed thoroughly with DI water between each measurement, and the probe was held in the gelling solutions for at least 30 s before measuring the value.

### Thermodynamic Constants by NMR

Van’t Hoff analysis experiments were taken using a JEOL 400 MHz nuclear magnetic resonance spectrometer. ^1^H NMR spectra were taken from 25 °C in 5 °C steps up to 80 °C for pre-GdL samples and 70 °C for post-GdL samples. A known concentration (set to 0.5 equiv relative to the LMWG in question) of an internal standard ((3-trimethylsilyl)propionic-2,2,3,3-d_4_ acid sodium salt) was used to assess the concentration of NMR visible LMWG as the temperature was increased. The samples were measured both before and after gelation via the GdL addition method detailed above except here with the use of deuterium oxide and sodium deuteroxide in the place of water and sodium hydroxide, respectively. All linear fits for post GdL gave *R*^2^ values greater than 0.98. It was seen that baseline correction functions were suitable for EDOT-GFF and EDOT-GFFD giving highly linear data in the ln(*S*) versus 1/*T* plots. In the case of EDOT-FFF, the exceptionally low solubilities meant that integrations below 40 °C were not usable. Since the gel phase appears to break down above 60 °C by rheology (Figure S6a), only the region of 40–60 °C was used to estimate the dissociation thermodynamics for EDOT-FFF.

As mentioned in the main text, it was assumed based on literature precedent^39^ that the LMWGs were either free in solution if NMR was visible or incorporated into the large, assembled macrostructures if NMR was invisible.

### Small-Angle Neutron Scattering

Measurements were performed at the ZOOM beamline of the ISIS pulsed neutron source at the Rutherford Appleton Laboratory (Didcot, U.K.). Measurements were taken both pre-GdL and greater than 12 h post-GdL addition. The assembly protocol was as described above except for the use of deuterium oxide in the place of water. Samples were measured for 10 μA at 25 °C. The pinhole collimation was set to L1 = L2 = 4 m while sample-detector distances were configured to give a scattering vector Q = (4π/λ)sin(θ/2) range of 0.004–0.722 Å^–1^, where θ is the scattering angle and neutrons of wavelengths (λ) of 1.75–16.5 Å were used simultaneously by time of flight. Data reduction was performed using MantidPlot,^70^ and the SANS curves were fitted with SasView v5.0.4.^55^ A solvent scattering length density (SLD) of 6.3 × 10^–6^ Å^–2^ was assumed and was calculated for the LMWG in question using SasView’s SLD calculator (1.71, 1.73, and 1.74 × 10^–6^ Å^–2^ for EDOT-FFF, EDOT-GFF, and EDOT-GFFD respectively). Background subtractions were performed using the mixtures minus the gelators.

### Molecular Dynamics Methodology

Atomic coordinates for the initial models of the five EDOT-peptide variants were constructed in extended conformations (*trans* peptide backbones) using Discovery Studio Visualizer.^56^ Systems with one, two, or 20 individual EDOT-peptide monomers were created using the Packmol^57^ program to place all constituent monomers at an initial separation distance > 1.5 nm. EDOT-peptides were then explicitly solvated in cubic simulation cells with side lengths of 5 nm (∼12,000 water molecules) and 9 nm (∼70,000 water molecules) for the systems containing one/two monomers and 20 monomers, respectively. All titratable groups were fully protonated to emulate low pH conditions, with Na^+^ and Cl^–^ counterions present at the experimentally relevant 15 mM concentration.

Molecular dynamics (MD) simulations were performed using the GROMACS 2023 software package^58^ with interatomic interactions for peptides described by the CHARMM36 protein force field^59^ and the EDOT moieties treated with the CHARMM General Force field (CGenFF, version 4.0).^60^ Atomic charges and missing parameters were assigned via analogy and optimized to be CHARMM-compatible using the Visual Molecular Dynamics (VMD) 1.9.4 software,^61^ the Force Field Toolkit (ffTK, version 2.0) plugin,^62^ and Gaussian 16 (revision C01)^63^ (see Tables S2 and S3). In all simulations, periodic boundary conditions were employed. Long-range electrostatics were treated with the Particle Mesh Ewald (PME)^64^ method with a 12 Å cutoff and 1.2 Å fast Fourier transform (FFT) grid spacing. van der Waals interactions had a cutoff distance of 12 Å with force switching at 10 Å. To avoid steric clashes in the starting atomic coordinates, energy minimization was performed using the steepest descent algorithm and an energy convergence criterion of 100 kJ mol^–1^. The Berendsen^65^ weak-coupling scheme was initially used to efficiently relax the system to the target temperature of 298 K and pressure of 1 atm. Equilibration was performed for 250 ps of MD in the canonical (NVT) ensemble with the EDOT-peptide monomers restrained to their initial positions, followed by 750 ps of unrestrained MD in the isothermal–isobaric (NPT) ensemble. Finally, the extended-ensemble Nosé–Hoover^66,67^ and Parrinello–Rahman^68^ coupling schemes were used to perform NPT simulations for data collection. An integration time step of 2 fs was used for all simulations, and the LINCS algorithm^69^ was employed to constrain the length of bonds containing hydrogen atoms.^69^ Each model system was simulated with five independent trajectories with an output frequency of 5 ps. Systems containing one or two EDOT-peptide monomers ran for 1 μs, with quantitative analysis reported on the last 100 ns. Those containing 20 EDOT-peptide monomers ran for 100 ns, with quantitative analysis reported on the last 10 ns. Solvent accessible surface area and hydrogen bond analysis was performed using GROMACS tools and qualitative analysis and rendering was performed using VMD 1.9.4 software.

### Four-Point Probe

Films were fabricated in rectangular PDMS molds with dimensions of 10 × 10 mm. Film thicknesses were assessed by imaging using an Ossila contact angle goniometer and comparing observed thicknesses, averaged across the film, with the thickness of the glass slide beneath (known to be 1 mm).

Gels were prepared in the PDMS molds for 24 h using the assembly protocol described above. These were placed in a sealed container during gelation with a Milli-Q water reservoir to prevent dehydration. Once unsealed, measurements were immediately taken using an Ossila four-point probe and thicknesses estimated. At least 3 measurements were made per sample for at least 3 samples per LMWG. The mean of the resulting conductivities and sheet resistances was calculated and stated in the main text. The error given is the standard deviation in each case, with the distribution here assumed to be normal.

### Electrochemical Impedance Spectroscopy (EIS)

EIS measurements were taken by using a Palmsens4 electrochemical interface and glassy carbon electrodes. Gels were prepared in custom-made cylindrical PDMS molds with diameters around 7 mm. After removal from the molds for measurement, the thickness was found to vary and so is accounted for in each case through measurement using a pair of calipers. Samples were measured using a frequency sweep from 10^6^ to 0.1 Hz with 20 measurements per decade, an AC voltage of 0.01 V and equilibration time of 10 s. Analysis and circuit fitting were undertaken using the Z-view (v2) software. Mean and error were calculated as for four-point probe. The electrolyte control consisted of simply the NaOH and GdL in Milli-Q water without the gelator, again left for at least 12 h to mimic the gelation protocol and allow comparable GdL hydrolysis.

## Data Availability

Research raw data supporting is available upon reasonable request from rdm-enquiries@imperial.ac.uk.

## Supporting Information Available

Extra information on experimental methodologies; schematics of synthetic protocols; photographs of gels; additional rheology data; additional UV–vis absorption data; additional SANS data; additional impedance spectroscopy data; and additional AFM data (PDF)

## References

[ref1] Chen C.; Bai X.; Ding Y.; Lee I.-S. Electrical stimulation as a novel tool for regulating cell behavior in tissue engineering. Biomater. Res. 2019, 23, 25 10.1186/s40824-019-0176-8. 31844552 PMC6896676

[ref2] Sun B.; Wu T.; Wang J.; Li D.; Wang J.; Gao Q.; Bhutto M.; El Hamshary H.; Al-Deyab S.; Mo X. Polypyrrole-coated poly(L-lactic acid-co-ε-caprolactone)/silk fibroin nanofibrous membranes promoting neural cell proliferation and differentiation with electrical stimulation. J. Mater. Chem. B 2016, 4, 6670–6679. 10.1039/C6TB01710J. 32263522

[ref3] Love M. R.; Palee S.; Chattipakorn S. C.; Chattipakorn N. Effects of electrical stimulation on cell proliferation and apoptosis. J. Cell. Physiol. 2018, 233, 1860–1876. 10.1002/jcp.25975. 28452188

[ref4] Hu M.; Hong L.; Liu C.; Hong S.; He S.; Zhou M.; Huang G.; Chen Q. Electrical stimulation enhances neuronal cell activity mediated by Schwann cell derived exosomes. Sci. Rep. 2019, 9, 4206 10.1038/s41598-019-41007-5. 30862846 PMC6414536

[ref5] Stoppel W. L.; Kaplan D. L.; Black L. D. Electrical and mechanical stimulation of cardiac cells and tissue constructs. Adv. Drug Delivery Rev. 2016, 96, 135–155. 10.1016/j.addr.2015.07.009. PMC4698182 26232525

[ref6] Smith A. M.; Williams R.; Tang C.; Coppo P.; Collins R.; Turner M.; Saiani A.; Ulijn R. Fmoc-Diphenylalanine Self Assembles to a Hydrogel via a Novel Architecture Based on π–π Interlocked β-Sheets. Adv. Mater. 2008, 20, 37–41. 10.1002/adma.200701221.

[ref7] Gazit E. Self Assembly of Short Aromatic Peptides into Amyloid Fibrils and Related Nanostructures. Prion 2007, 1, 32–35. 10.4161/pri.1.1.4095. 19164892 PMC2633705

[ref8] Wojciechowski J. P.; Martin A. D.; Thordarson P. Kinetically Controlled Lifetimes in Redox-Responsive Transient Supramolecular Hydrogels. J. Am. Chem. Soc. 2018, 140, 2869–2874. 10.1021/jacs.7b12198. 29406709

[ref9] McAulay K.; Thomson L.; Porcar L.; Schweins R.; Mahmoudi N.; Adams D. J.; Draper E. R. Using Rheo-Small-Angle Neutron Scattering to Understand How Functionalised Dipeptides Form Gels. Org. Mater. 2020, 02, 108–115. 10.1055/s-0040-1708832.

[ref10] Draper E. R.; Eden E. G. B.; McDonald T. O.; Adams D. J. Spatially resolved multicomponent gels. Nat. Chem. 2015, 7, 848–852. 10.1038/nchem.2347. 26391086

[ref11] Awhida S.; Draper E. R.; McDonald T. O.; Adams D. J. Probing gelation ability for a library of dipeptide gelators. J. Colloid Interface Sci. 2015, 455, 24–31. 10.1016/j.jcis.2015.05.032. 26047582

[ref12] Chen L.; Revel S.; Morris K.; Adams D. J. Energy transfer in self-assembled dipeptide hydrogels. Chem. Commun. 2010, 46, 4267–4269. 10.1039/c003052j. 20461244

[ref13] Sanders A. M.; Magnanelli T. J.; Bragg A. E.; Tovar J. D. Photoinduced Electron Transfer within Supramolecular Donor–Acceptor Peptide Nanostructures under Aqueous Conditions. J. Am. Chem. Soc. 2016, 138, 3362–3370. 10.1021/jacs.5b12001. 26900714

[ref14] Ardoña H. A. M.; Tovar J. D. Energy transfer within responsive pi-conjugated coassembled peptide-based nanostructures in aqueous environments. Chem. Sci. 2015, 6, 1474–1484. 10.1039/C4SC03122A. 29560236 PMC5811113

[ref15] Hulvat J. F.; Sofos M.; Tajima K.; Stupp S. I. Self-Assembly and Luminescence of Oligo(*p* -phenylene vinylene) Amphiphiles. J. Am. Chem. Soc. 2005, 127, 366–372. 10.1021/ja047210m. 15631487

[ref16] Prasanthkumar S.; Saeki A.; Seki S.; Ajayaghosh A. Solution Phase Epitaxial Self-Assembly and High Charge-Carrier Mobility Nanofibers of Semiconducting Molecular Gelators. J. Am. Chem. Soc. 2010, 132, 8866–8867. 10.1021/ja103685j. 20536178

[ref17] Yagai S.; Kinoshita T.; Kikkawa Y.; Karatsu T.; Kitamura A.; Honsho Y.; Seki S. Interconvertible Oligothiophene Nanorods and Nanotapes with High Charge-Carrier Mobilities. Chem. Eur. J. 2009, 15, 9320–9324. 10.1002/chem.200901336. 19637167

[ref18] Mukherjee A.; Sakurai T.; Seki S.; Ghosh S. Ultrathin Two Dimensional (2D) Supramolecular Assembly and Anisotropic Conductivity of an Amphiphilic Naphthalene-Diimide. Langmuir 2020, 36, 13096–13103. 10.1021/acs.langmuir.0c02604. 33103440

[ref19] Ing N. L.; Spencer R. K.; Luong S. H.; Nguyen H. D.; Hochbaum A. I. Electronic Conductivity in Biomimetic α-Helical Peptide Nanofibers and Gels. ACS Nano 2018, 12, 2652–2661. 10.1021/acsnano.7b08756. 29537817

[ref20] James E. I.; Jenkins L. D.; Murphy A. R. Peptide-Thiophene Hybrids as Self-Assembling Conductive Hydrogels. Macromol. Mater. Eng. 2019, 304, 1900285 10.1002/mame.201900285.

[ref21] Blatz T. J.; Fry M.; James E.; Albin T.; Pollard Z.; Kowalczyk T.; Murphy A. R. Templating the 3D structure of conducting polymers with self-assembling peptides. J. Mater. Chem. B 2017, 5, 4690–4696. 10.1039/C7TB00221A. 32264311

[ref22] Draper E. R.; Adams D. J. Controlling the Assembly and Properties of Low-Molecular-Weight Hydrogelators. Langmuir 2019, 35, 6506–6521. 10.1021/acs.langmuir.9b00716. 31038973

[ref23] Reches M.; Gazit E. Self-assembly of peptide nanotubes and amyloid-like structures by charged-termini-capped diphenylalanine peptide analogues. Isr. J. Chem. 2005, 45, 363–371. 10.1560/5MC0-V3DX-KE0B-YF3J.

[ref24] Frederix P. W. J. M.; Scott G.; Abul-Haija Y.; Kalafatovic D.; Pappas C.; Javid N.; Hunt N.; Ulijn R.; Tuttle T. Exploring the sequence space for (tri-)peptide self-assembly to design and discover new hydrogels. Nat. Chem. 2015, 7, 30–37. 10.1038/nchem.2122. 25515887

[ref25] Tang C.; Ulijn R. V.; Saiani A. Effect of Glycine Substitution on Fmoc–Diphenylalanine Self-Assembly and Gelation Properties. Langmuir 2011, 27, 14438–14449. 10.1021/la202113j. 21995651

[ref26] Abul-Haija Y. M.; Scott G. G.; Sahoo J. K.; Tuttle T.; Ulijn R. V. Cooperative, ion-sensitive co-assembly of tripeptide hydrogels. Chem. Commun. 2017, 53, 9562–9565. 10.1039/C7CC04796G. 28805225

[ref27] Spicer C. D.; Booth M.; Mawad D.; Armgarth A.; Nielsen C.; Stevens M. M. Synthesis of Hetero-bifunctional, End-Capped Oligo-EDOT Derivatives. Chem. 2017, 2, 125–138. 10.1016/j.chempr.2016.12.003. 28149959 PMC5268340

[ref28] Adams D. J.; Butler M. F.; Frith W. J.; Kirkland M.; Mullen L.; Sanderson P. A new method for maintaining homogeneity during liquid-hydrogel transitions using low molecular weight hydrogelators. Soft Matter 2009, 5, 1856–1862. 10.1039/b901556f.

[ref29] Martin A. D.; Wojciechowski J. P.; Warren H.; in het Panhuis M.; Thordarson P. Effect of heterocyclic capping groups on the self-assembly of a dipeptide hydrogel. Soft Matter 2016, 12, 2700–2707. 10.1039/C6SM00025H. 26860207

[ref30] Gupta J. K.; Adams D. J.; Berry N. G. Will it gel? Successful computational prediction of peptide gelators using physicochemical properties and molecular fingerprints. Chem. Sci. 2016, 7, 4713–4719. 10.1039/C6SC00722H. 30155120 PMC6016447

[ref31] Bianco S.; Panja S.; Adams D. J. Using Rheology to Understand Transient and Dynamic Gels. Gels 2022, 8, 132 10.3390/gels8020132. 35200514 PMC8872063

[ref32] Park S.; Aglyamov S.; Scott W.; Emelianov S.; Sethuraman S.; Rubin J.; Shah J.; Karpiouk A.; Mallidi S.; Smalling R. 1E-5 Synergy and Applications of Combined Ultrasound, Elasticity, and Photoacoustic Imaging (Invited). In 2006 IEEE Ultrasonics Symposium; IEEE: 2006; pp 405–415 10.1109/ULTSYM.2006.114.

[ref33] Draper E. R.; Dietrich B.; McAulay K.; Brasnett C.; Abdizadeh H.; Patmanidis I.; Marrink S.; Su H.; Cui H.; Schweins R.; et al. Using Small-Angle Scattering and Contrast Matching to Understand Molecular Packing in Low Molecular Weight Gels. Matter 2020, 2, 764–778. 10.1016/j.matt.2019.12.028.

[ref34] Martinez C. R.; Iverson B. L. Rethinking the term “pi-stacking. Chem. Sci. 2012, 3, 2191–2201. 10.1039/c2sc20045g.

[ref35] By Kasha theory a red shift might imply H-like stacking while a blue shift might suggest J-like stacking. It should be noted, in general, Kasha theory only considers coulomb coupling and does not account for the intricate vibronic landscape found in many chromophores.

[ref36] Hestand N. J.; Spano F. C. Expanded Theory of H- and J-Molecular Aggregates: The Effects of Vibronic Coupling and Intermolecular Charge Transfer. Chem. Rev. 2018, 118, 7069–7163. 10.1021/acs.chemrev.7b00581. 29664617

[ref37] Raeburn J.; Mendoza-Cuenca C.; Cattoz B. N.; Little M. A.; Terry A. E.; Zamith Cardoso A.; Griffiths P. C.; Adams D. J. The effect of solvent choice on the gelation and final hydrogel properties of Fmoc–diphenylalanine. Soft Matter 2015, 11, 927–935. 10.1039/C4SM02256D. 25516486

[ref38] Fuentes-Caparrós A. M.; McAulay K.; Rogers S. E.; Dalgliesh R. M.; Adams D. J. On the Mechanical Properties of N-Functionalised Dipeptide Gels. Molecules 2019, 24, 3855 10.3390/molecules24213855. 31731551 PMC6864704

[ref39] Hirst A. R.; Coates I.; Boucheteau T.; Miravet J.; Escuder B.; Castelletto V.; Hamley I.; Smith D. Low-Molecular-Weight Gelators: Elucidating the Principles of Gelation Based on Gelator Solubility and a Cooperative Self-Assembly Model. J. Am. Chem. Soc. 2008, 130, 9113–9121. 10.1021/ja801804c. 18558681

[ref40] Draper E. R.; Su H.; Brasnett C.; Poole R.; Rogers S.; Cui H.; Seddon A.; Adams D. J. Opening a Can of Worm(-like Micelle)s: The Effect of Temperature of Solutions of Functionalized Dipeptides. Angew. Chem., Int. Ed. 2017, 56, 10467–10470. 10.1002/anie.201705604. PMC5577516 28653804

[ref41] Bai S.; Pappas C.; Debnath S.; Frederix P. W. J. M.; Leckie J.; Fleming S.; Ulijn R. V. Stable Emulsions Formed by Self-Assembly of Interfacial Networks of Dipeptide Derivatives. ACS Nano 2014, 8, 7005–7013. 10.1021/nn501909j. 24896538

[ref42] Yoshii T.; Onogi S.; Shigemitsu H.; Hamachi I. Chemically Reactive Supramolecular Hydrogel Coupled with a Signal Amplification System for Enhanced Analyte Sensitivity. J. Am. Chem. Soc. 2015, 137, 3360–3365. 10.1021/ja5131534. 25679407

[ref43] Han T. H.; Ok T.; Kim J.; Shin D. O.; Ihee H.; Lee H.; Kim S. O. Bionanosphere Lithography via Hierarchical Peptide Self-Assembly of Aromatic Triphenylalanine. Small 2010, 6, 945–951. 10.1002/smll.200902050. 20397209

[ref44] Shmilovich K.; Mansbach R. A.; Sidky H.; Dunne O. E.; Panda S. S.; Tovar J. D.; Ferguson A. L. Discovery of Self-Assembling π-Conjugated Peptides by Active Learning-Directed Coarse-Grained Molecular Simulation. J. Phys. Chem. B 2020, 124, 3873–3891. 10.1021/acs.jpcb.0c00708. 32180410

[ref45] Yeh M.-Y.; Huang C.; Lai T.; Chen F.; Chu N.; Tseng D. T.; Hung S.; Lin H. Effect of Peptide Sequences on Supramolecular Interactions of Naphthaleneimide/Tripeptide Conjugates. Langmuir 2016, 32, 7630–7638. 10.1021/acs.langmuir.6b01809. 27385634

[ref46] Wang H.; Wang Z.; Yi X.; Long J.; Liu J.; Yang Z. Anti-degradation of a recombinant complex protein by incoporation in small molecular hydrogels. Chem. Commun. 2011, 47, 955–957. 10.1039/C0CC04249H. 21079842

[ref47] Williams-Noonan B. J.; Kamboukos A.; Todorova N.; Yarovsky I. Self-assembling peptide biomaterials: Insights from spontaneous and enhanced sampling molecular dynamics simulations. Chem. Phys. Rev. 2023, 4, 021304 10.1063/5.0142302.

[ref48] Lin Y.; Penna M.; Spicer C. D.; Higgins S. G.; Gelmi A.; Kim N.; Wang S.; Wojciechowski J. P.; Pashuck E. T.; Yarovsky I.; Stevens M. M. High-Throughput Peptide Derivatization toward Supramolecular Diversification in Microtiter Plates. ACS Nano 2021, 15, 4034–4044. 10.1021/acsnano.0c05423. 33587607 PMC7992134

[ref49] Lin Y.; Penna M.; Thomas M. R.; Wojciechowski J. P.; Leonardo V.; Wang Y.; Pashuck E. T.; Yarovsky I.; Stevens M. M. Residue-Specific Solvation-Directed Thermodynamic and Kinetic Control over Peptide Self-Assembly with 1D/2D Structure Selection. ACS Nano 2019, 13, 1900–1909. 10.1021/acsnano.8b08117. 30673202 PMC6396410

[ref50] See the Nyquist plot in Figure 8d, where the measurement frequency ω → 0, and the impedance does not tend to an intercept with the real *x*-axis.

[ref51] Feig V. R.; Tran H.; Bao Z. Biodegradable polymeric materials in degradable electronic devices. ACS Cent. Sci. 2018, 4, 337–348. 10.1021/acscentsci.7b00595. 29632879 PMC5879474

[ref52] Mech-Dorosz A.; Khan M.; Mateiu R.; Hélix-Nielsen C.; Emnéus J.; Heiskanen A. Impedance characterization of biocompatible hydrogel suitable for biomimetic lipid membrane applications. Electrochim. Acta 2021, 373, 137917 10.1016/j.electacta.2021.137917.

[ref53] An “open” Warburg element is an Warburg element in the limit where a finite boundary layer (region of concentration different from the bulk) exists and is space limited or contains a reflective boundary. This means the system will tend toward a capacitor as 0 frequency is approached since no current passes through the electrode interface.

[ref54] Jorcin J.-B.; Orazem M. E.; Pébère N.; Tribollet B. CPE analysis by local electrochemical impedance spectroscopy. Electrochim. Acta 2006, 51, 1473–1479. 10.1016/j.electacta.2005.02.128.

[ref70] Arnold O.; Bilheux J.C.; Borreguero J.M.; Buts A.; Campbell S.I.; Chapon L.; Doucet M.; Draper N.; Ferraz Leal R.; Gigg M.A.; Lynch V.E.; Markvardsen A.; Mikkelson D.J.; Mikkelson R.L.; Miller R.; Palmen K.; Parker P.; Passos G.; Perring T.G.; Peterson P.F.; Ren S.; Reuter M.A.; Savici A.T.; Taylor J.W.; Taylor R.J.; Tolchenov R.; Zhou W.; Zikovsky J. Mantid—Data analysis and visualization package for neutron scattering and μ SR experiments. Nucl. Instrum. Methods Phys. Res., Sect. A 2014, 764, 156–166. 10.1016/j.nima.2014.07.029.

[ref55] Doucet M.; Cho J. H.; Alina G.; Attala Z.; Bakker J.; Bouwman W.; Butler P.; Campbell K.; Cooper-Benun T.; Durniak C.; SasView 5.0.4. Preprint, 2021 10.5281/zenodo.4467703.

[ref56] BIOVIA Discovery Studio Visualizer, Release 3.5; Dassault Systèmes: San Diego, 2012.

[ref57] Martínez L.; Andrade R.; Birgin E. G.; Martínez J. M. PACKMOL : A package for building initial configurations for molecular dynamics simulations. J. Comput. Chem. 2009, 30, 2157–2164. 10.1002/jcc.21224. 19229944

[ref58] Abraham M. J.; Murtola T.; Schulz R.; Páll S.; Smith J.; Hess B.; Lindahl E. GROMACS: High performance molecular simulations through multi-level parallelism from laptops to supercomputers. SoftwareX 2015, 1–2, 19–25. 10.1016/j.softx.2015.06.001.

[ref59] Best R. B.; Zhu X.; Shim J.; Lopes P. E. M.; Mittal J.; Feig M.; MacKerell A. D. Optimization of the Additive CHARMM All-Atom Protein Force Field Targeting Improved Sampling of the Backbone ϕ, ψ and Side-Chain χ _1_ and χ _2_ Dihedral Angles. J. Chem. Theory Comput. 2012, 8, 3257–3273. 10.1021/ct300400x. 23341755 PMC3549273

[ref60] Vanommeslaeghe K.; MacKerell A. D. Automation of the CHARMM General Force Field (CGenFF) I: Bond Perception and Atom Typing. J. Chem. Inf. Model. 2012, 52, 3144–3154. 10.1021/ci300363c. 23146088 PMC3528824

[ref61] Humphrey W.; Dalke A.; Schulten K. VMD: Visual molecular dynamics. J. Mol. Graph 1996, 14, 33–38. 10.1016/0263-7855(96)00018-5. 8744570

[ref62] Mayne C. G.; Saam J.; Schulten K.; Tajkhorshid E.; Gumbart J. C. Rapid parameterization of small molecules using the force field toolkit. J. Comput. Chem. 2013, 34, 2757–2770. 10.1002/jcc.23422. 24000174 PMC3874408

[ref63] Frisch M. J.; Trucks G. W.; Schlegel H. B.; Scuseria G. E.; Robb M. A.; Cheeseman J. R.; Scalmani G.; Barone V.; Petersson G. A.; Nakatsuji H.; Gaussian 16; Gaussian: Wallingford, CT, 2016.

[ref64] Essmann U.; Perera P.; Berkowitz M. L.; Darden T.; Lee H.; Pedersen L. G. A smooth particle mesh Ewald method. J. Chem. Phys. 1995, 103, 8577–8593. 10.1063/1.470117.

[ref65] Berendsen H. J. C.; Postma J. P. M.; van Gunsteren W. F.; DiNola A.; Haak J. R. Molecular dynamics with coupling to an external bath. J. Chem. Phys. 1984, 81, 3684–3690. 10.1063/1.448118.

[ref66] Nosé S. A molecular dynamics method for simulations in the canonical ensemble. Mol. Phys. 1984, 52, 255–268. 10.1080/00268978400101201.

[ref67] Hoover W. G. Canonical dynamics: Equilibrium phase-space distributions. Phys. Rev. A 1985, 31, 1695–1697. 10.1103/PhysRevA.31.1695. 9895674

[ref68] Parrinello M.; Rahman A. Polymorphic transitions in single crystals: A new molecular dynamics method. J. Appl. Phys. 1981, 52, 7182–7190. 10.1063/1.328693.

[ref69] Hess B.; Bekker H.; Berendsen H. J. C.; Fraaije J. G. E. M. LINCS: A linear constraint solver for molecular simulations. J. Comput. Chem. 1997, 18, 1463–1472. 10.1002/(SICI)1096-987X(199709)18:12<1463::AID-JCC4>3.0.CO;2-H.

