# Supplementary material for: 3,4-Ethylenedioxythiophene Hydrogels: Relating Structure and Charge Transport in Supramolecular Gels
Source: Chem Mater. 2024 Mar 25;36(7):3092–106. doi: 10.1021/acs.chemmater.3c01360 (PMC11007859; doi:10.1021/acs.chemmater.3c01360)
Supplement: Supplementary file 1 — cm3c01360_si_001.pdf [file cm3c01360_si_001.pdf]

Supplementary information for: 3,4-Ethylenedioxythiophene Hydrogels: Relating Structure and Charge Transport in Supramolecular Gels

Luke C. B. Salter, [a] *Jonathan P. Wojciechowski,* [a] *Ben McLean,*[b,c] *Patrick Charchar,*[b] *Piers R. F. Barnes,*[d] *Adam Creamer,* [a] *James Doutch,*[e] *Hanna M. G. Barriga,*[f] *Margaret N. Holme,*[f] *Irene Yarovsky,*[b] *and Molly M. Stevens**[a,f,g]

[a]Department of Materials, Department of Bioengineering, Institute of Biomedical Engineering, Imperial College London, London, SW7 2AZ, United Kingdom.

[b]School of Engineering, RMIT University, Melbourne, Victoria 3001, Australia.

[c]ARC Research Hub for Australian Steel Innovation

[d]Department of Physics, Imperial College London, London, SW7 2AZ, United Kingdom.

[e]ISIS Muon and Neutron Source, Rutherford Appleton Laboratory, Harwell Campus, Oxfordshire, OX11 0QX, United Kingdom

[f]Department of Medical Biochemistry and Biophysics, Karolinska Institute, 171 77 Stockholm, Sweden

[g]Department of Physiology, Anatomy and Genetics, Department of Engineering Science, and Kavli Institute for Nanoscience Discovery, University of Oxford, OX1 3QU, Oxford, United Kingdom

Table of Contents

Synthesis and chemical characterisation 3

EDOT-peptide synthesis general procedure - (iii) in Figure S1. 4

High-performance liquid chromatography (HPLC) purification 6

Chemical analysis 6

Supplementary figures 11

NMR spectra 26

References 32

Supplementary information

Synthesis and chemical characterisation

The synthesis of each peptide was undertaken as shown in the scheme below.

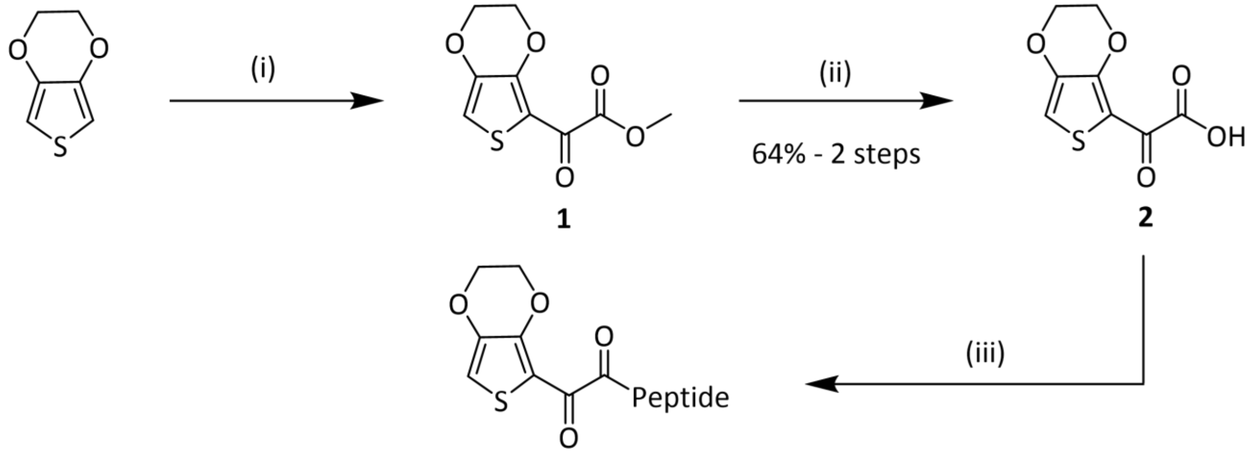

Figure S1. Synthetic scheme for synthesis of the EDOT-peptide molecules in this study. Conditions: (i) EDOT, oxalyl chloride, 100 °C, MeOH, triethylamine, room temperature; (ii) THF, NaOH, room temperature, 2 hours; (iii) Solid phase peptide synthesis (see text below).

2-(2,3-dihydrothieno[3,4-b][1,4]dioxin-5-yl)-2-oxoacetic acid (**2**):

Synthesis of **1** and **2** was undertaken as reported previously by Spicer *et al.*[1] giving the desired product as a yellow solid with a yield of 64% over two steps.

In brief, oxalyl chloride (2.55 mL, 30 mmol, 1 eq.) was added in a drop-wise fashion to a solution of EDOT (3.15 mL, 30 mmol, 1 eq.) in dioxane (150 mL). The solution was heated to 100 ° C for 1 hour stirring constantly. Once cooled to room temperature, methanol (90 mL) was added along with triethylamine (5 eq.) following which the mixture was stirred for a further 3 hours. Excess alcohol was then removed in vacuo. The mixture was then diluted with dichloromethane (300 mL), washed with water (100 mL) and brine (100 mL), the separated organic layers dried over $MgSO_4$, filtered and concentrated in vacuo. The resulting residue was then purified by column chromatography using 50% (v/v) ethyl acetate in hexane to yield the desired methyl ester as a yellow solid. This ester was then dissolved in THF (100 mL), then an excess of 1 M aqueous sodium hydroxide (> 5 eq) was added and the reaction mixture stirred for 2 hours at room temperature. Excess tetrahydrofuran was removed in vacuo, then the mixture was acidified with 1 M aqueous hydrochloric acid solution (100 mL) the precipitate was centrifuged and washed with DI water (3 x 100 mL). The solid was dried by lyophilisation yielding the desired oxalyl acid in a 64% yield over 2 steps. Spectroscopic data was consistent with those reported previously.[1] **^{1}H NMR** (400 MHz, $CDCl_3$): δ = 7.35 (1H, s, -S-C<u>H</u>), 4.38 (2H, m, -O-C<u>H2</u>-), 4.27 (2H, m, -O-C<u>H2</u>).

EDOT-peptide synthesis general procedure - (iii) in Figure S1.

Peptide synthesis was achieved either manually or using a Biotage Initiator Alstra+ peptide synthesiser.

Peptides were constructed using conventional Fmoc solid-phase peptide synthesis techniques and capped at the N-terminus with the requisite EDOT oligomer. In brief the resin was swelled in dichloromethane (DCM) (5 mL) inside a capped and fritted syringe. This was expelled and replaced with a solution of the amino acid (3 eq.) and *N,N*-diisopropylethylamine (DIPEA) (6 eq.)

the estimated amount of available chlorotrityl chloride on the resin being designated as 1 eq. This was shaken overnight after which the mixture was expelled, the resin washed 5x with DCM (5 mL), 5x with dimethylformamide (DMF) (5 mL) followed by shaking with 3 mL of a 9:1:0.5 (v/v) DCM:MeOH:DIPEA solution (5 mL) for 20 minutes. If the peptide synthesizer was used this was the point at which they would be loaded after a further 5x DCM, 5x DMF (5 mL each) wash. Samples would need only be cleaved after automated synthesis (see cleavage protocol below). To cleave Fmoc groups on the N-terminus the mixture was expelled, the resin again washed 5x with DCM and DMF (5 mL) respectively and then shaken with 20% piperidine in DMF (3 mL) for 1 minute. This was expelled and replaced again with 20% piperidine in DMF (3 mL) for 20 minutes. To couple amino acids to the free amine, the resin was again washed 5x with DCM and DMF (5 mL) respectively following which a 2-3 mL solution in dry DMF of the amino acid (3 eq.), DIPEA (6 eq.) and HATU (0.95 eq.) (5 mL) was taken up and shaken for between 30-60 minutes. The above cleavage and coupling procedures were repeated as many times as necessary to build the required peptide. Between each step a Kaiser test was performed to check for coupling and cleavage success. When coupling the final EDOT capping group the same procedure was used as described above only often with < 3 eq. depending on availability.

To cleave the EDOT-peptides from the resin a solution of 20% trifluoroacetic acid (TFA) in DCM (3 mL) was taken up and shaken for 15-20 minutes. In the case where an aspartic acid was present, a 95:2.5:2.5 (v/v) TFA:water:triisopropylsilane solution (3 mL) was used instead. The solution was transferred into a round-bottom flask (RBF) and concentrated on a rotary evaporator with the assistance of DCM as an azeotrope to remove the TFA if needed.

High-performance liquid chromatography (HPLC) purification

Purification of the low molecular weight gelators (LMWGs) was undertaken using a Shimadzu Prominence LC-20A preparative HPLC with a flow rate of 10 mL/minute. The concentrated crude reaction mixture was dissolved in 40 mL 45-50% (v/v) acetonitrile (MeCN) in water (+ 0.1% (v/v) formic acid). The crude mixture was filtered through a 0.45 μm Teflon filter followed by injection onto the HPLC and eluted with a gradient of either 45-80 B% or 50-80 B% acetonitrile in water (+ 0.1% (v/v) formic acid). Fractions were collected, excess MeCN removed under reduced pressure and the mixture lyophilized to afford the desired products.

Chemical analysis

EDOT-COOH:

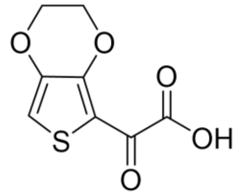

Isolated as a yellow solid in a yield of 79 %. NMR consistent with previous literature.[1] **^{1}H NMR** (400 MHz, d_6-DMSO): δ =7.35 (1H, s), 4.41 – 4.37 (2H, m), 4.30 – 4.26 (2H, m).

EDOT-FF:

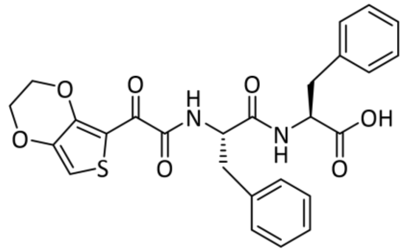

Isolated as a pale, yellow solid in a yield of 10%. **^{1}H NMR** (400 MHz, d_6-DMSO): δ = 8.46 (1H, d, NH, *J* = 8.7 Hz), 8.25 (1H, d, NH, *J* = 8.0 Hz), 7.23 – 7.10 (11H, m, phenyl ring protons and thiophene proton), 4.61 – 4.53 (1H, m, NH-CH), 4.49 – 4.43 (1H, m, NH-CH), 4.25 – 4.22 (2H, m, EDOT -O-CH_2), 4.20 – 4.16 (2H, m, EDOT -O-CH_2), 3.09 – 2.73 (4H, m, CH_2-Ph) ppm. **^{13}C NMR** (75 MHz, d_6-DMSO): δ = 177.05, 172.97, 170.50, 162.49, 150.04, 141.96, 137.89, 137.82, 129.68, 128.68, 128.56, 126.95, 126.85, 114.55, 111.22, 65.76, 64.27, 54.33, 54.05, 37.71, 37.40, 29.56. **HRMS** (Q-TOF) *m/z*: Calculated: 509.1377 $[M+H]^+$; Found: 509.1375 $[M+H]^+$

EDOT-FFD:

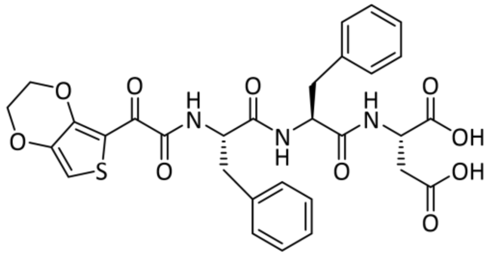

Isolated as a pale, yellow solid in a yield of 10%. **^{1}H NMR** (400 MHz, d_6-DMSO): δ = 8.59 (1H, d, NH, *J* = 8.8 Hz), 8.49 (1H, d, NH, *J* = 7.9 Hz), 8.33 (1H, d, NH, *J* = 8.5 Hz), 7.31 – 7.13 (11H, br, PhH and thiophene H), 4.62 (1H, td, NH-CH, *J* = 9.0, 4.6 Hz), 4.59 – 4.52 (2H, m, NH-CH),

4.29 – 4.27 (2H, m, -O-CH2), 4.23 - 4.20 (2H, m, -O-CH2), 3.10 - 2.77 (4H, m, Ph-CH2), 2.76 – 2.58 (2H, m, CO-CH2) ppm. **^{13}C NMR** (75 MHz, d_6-DMSO): δ = 206.76, 177.09, 170.42, 162.46, 150.01, 141.95, 138.21, 137.90, 129.77, 128.53, 126.80, 114.51, 111.25, 65.75, 64.27, 54.59, 54.35, 38.00, 37.74 ppm. **HRMS** (Q-TOF) *m/z*: Calculated: 624.1646 $[M+H]^+$; Found: 624.1638 $[M+H]^+$

EDOT-GFF:

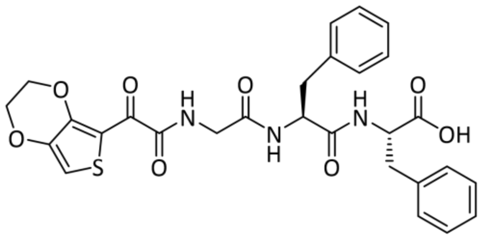

Isolated as a pale, yellow solid in a yield of 18%. **^{1}H NMR** (400 MHz, d_6-DMSO): δ = 8.74 (1H, t, NH (glycine), *J* = 6.0 Hz), 8.37 (1H, d, NH, *J* = 7.8 Hz), 8.07 (1H, d, NH, *J* = 8.5 Hz), 7.29-7.02 (11H, m, phenyl ring and thiophene protons), 4.56 (1H, td, NH-CH, *J* = 9.0. 4.3 Hz), 4.43 (1H, m, NH-CH, *J* = 8.4, 5.4 Hz), 4.38-4.31 (2H, m, EDOT -O-CH2), 4.27-4.20 (2H, m, EDOT -O-CH2) 3.82-3.62 (2H, m, NH-CH2), 3.11-2.68 (4H, m, CH2-Ph) ppm. **^{13}C NMR** (75 MHz, d_6-DMSO): δ = 192.03, 162.91, 155.78, 152.22, 150.08, 129.75, 129.63, 128.68, 128.49, 114.61, 65.63, 64.44, 53.99. **HRMS** (Q-TOF) *m/z*: Calculated: 566.1592 $[M+H]^+$; Found: 566.1606 $[M+H]^+$

EDOT-GFFD:

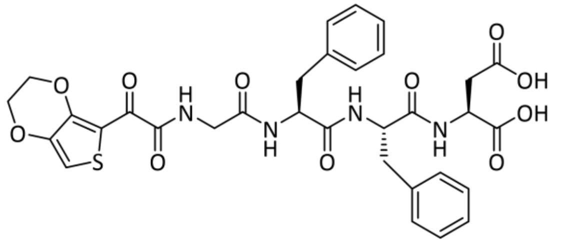

Isolated as a pale, yellow solid in a yield of 26%. **^{1}H NMR** (400 MHz, d_6-DMSO): δ = 8.74 (1H, t, N<u>H</u> (glycine), *J* = 6.1 Hz), 8.33 (1H, d, N<u>H</u>, *J* = 7.9 Hz), 8.23 (1H, d, N<u>H</u>, *J* = 8.4 Hz), 8.04 (1H, d, N<u>H</u>, *J* = 8.4 Hz), 7.32 – 7.07 (11H, m, phenyl ring and thiophene protons), 4.59 – 4.48 (3H, m, NH-C<u>H</u>), 4.37 – 4.32 (2H, m, EDOT -O-C<u>H$_2$</u>), 4.26 – 4.21 (2H, m, EDOT -O-C<u>H$_2$</u>), 3.81 – 3.63 (2H, m, NH-C<u>H$_2$</u>), 3.11 – 2.55 (6H, m, C<u>H$_2$</u>-COOH and C<u>H$_2$</u>-Ph) ppm. **^{13}C NMR** (75 MHz, d_6-DMSO): δ = 207.06, 172.48, 171.85, 171.17, 167.96, 162.51, 150.48, 138.12, 129.82, 129.76, 128.58, 128.49, 126.80, 114.90, 65.47, 64.23, 38.05, 31.22 ppm. **HRMS** (Q-TOF) *m/z*: Calculated: 681.1861 $[M+H]^+$; Found: 681.1868 $[M+H]^+$

EDOT-FFF:

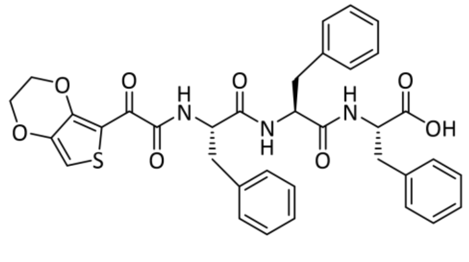

Isolated as a pale, yellow solid in a yield of 17%. **^{1}H NMR** (400 MHz, d_6-DMSO): δ = 8.61 (1H, d, N<u>H</u>, *J* = 8.9 Hz), 8.38 (1H, d, N<u>H</u>, *J* = 7.8 Hz), 8.26 (1H, d, N<u>H</u>, *J* = 8.5 Hz) 7.32-7.08 (16H, m

, Phenyl ring and thiophene protons), 4.60 (1H, td, NH-C_H_, *J* = 9.0, 4.5 Hz), 4.55-4.44 (2H, m, NH-C_H_, presumed two td overlapping, J values not clear), 4.33-4.15 (4H, m, O-C$\underline{H_2}$), 3.11-2.73 (6H, m, NH-CH-$\underline{CH_2}$) ppm. **^{13}C NMR** (75 MHz, d_6-DMSO): δ = 177.00, 170.85, 170.27, 162.38, 150.05, 141.94, 138.54, 138.19, 137.94, 129.76, 128.49, 126.61, 114.55, 111.19, 65.74, 64.26, 54.39, 38.21, 37.55 ppm. **HRMS** (Q-TOF) *m/z*: Calculated: 656.2061 $[M+H]^+$; Found: 656.2070 $[M+H]^+$

Fmoc-GFF:

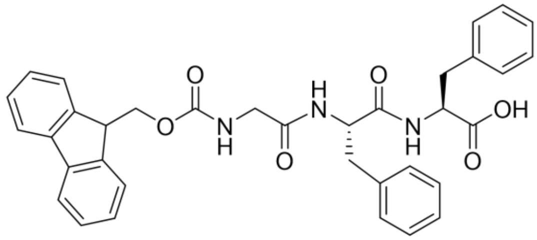

Isolated as a white solid in a yield of 36%. ^{1}H NMR consistent with those previously reported.[2]
^{1}H NMR (400 MHz, d_6-DMSO): δ = 8.33 (1H, d, J = 7.8 Hz), 7.95 (1H, d, J = 8.4 Hz), 7.89 (2H, d, J = 7.5 Hz), 7.70 (2H, d, J = 7.5 Hz), 7.46 (1H, t, J = 6.1 Hz), 7.44 – 7.38 (2H, m), 7.31 (2H, t, J = 7.4 Hz), 7.28 – 7.11 (10H, m), 4.55 (1H, td, J = 8.8, 4.2 Hz), 4.46 – 4.37 (1H, m), 4.27 – 2.17 (3H, m), 3.55 (2H, ddd, J = 44.3, 16.0, 5.3 Hz), 3.10 – 2.62 (4H, m).

Supplementary figures

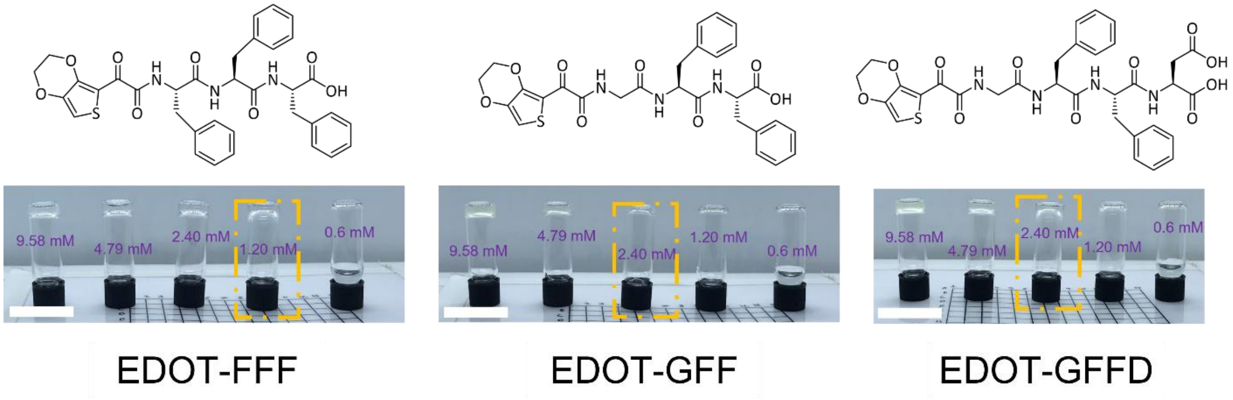

Figure S2. Photographs of the apparent minimum gel concentration by vial inversion test. Note this is not an accurate assessment but indicates the rough concentration range the materials were self-supporting down to. Scale bar = 2 cm.

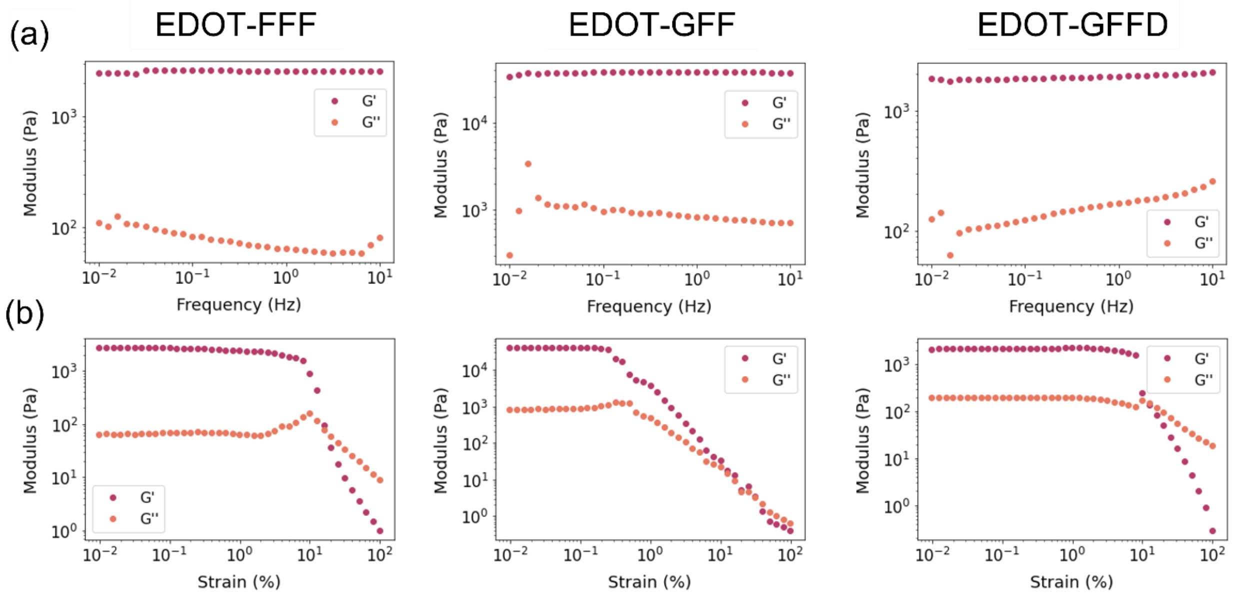

Figure S3. (a) Frequency and (b) strain sweeps of the three gelators validating the parameters used in our rheometry studies. Measurements taken on gels at a concentration of 10 mM.

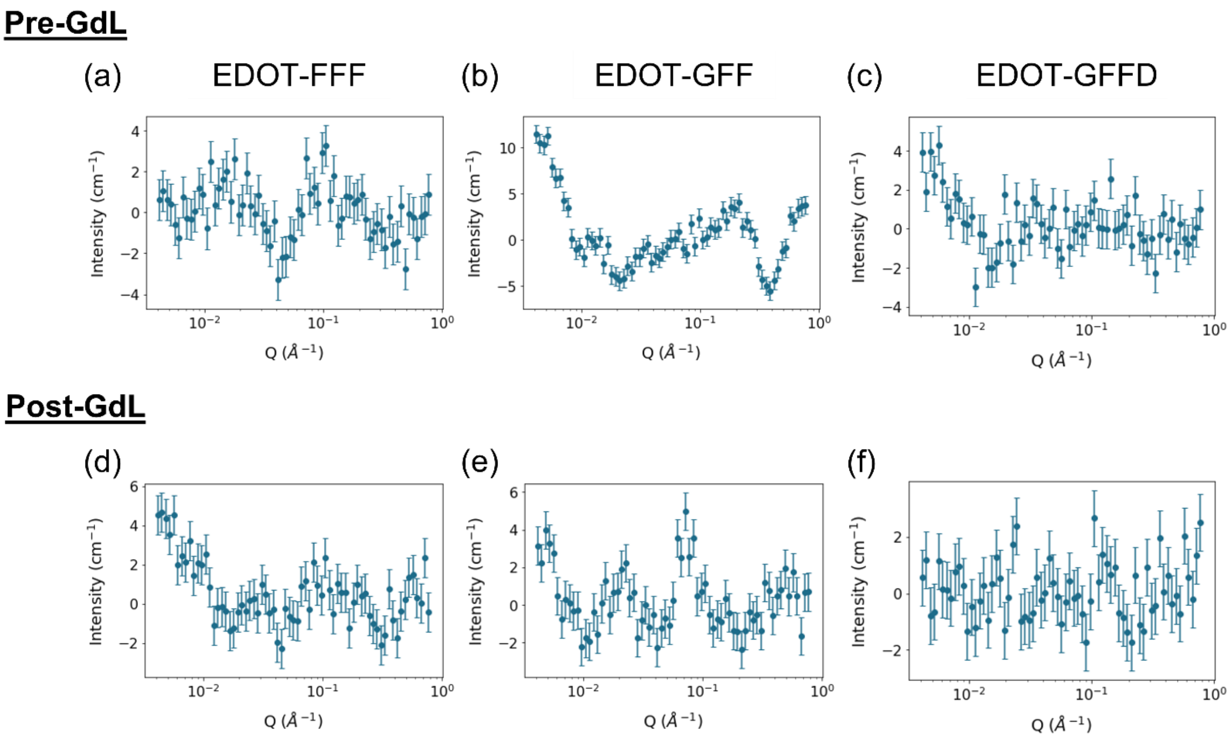

Figure S4. Plots of the residuals generated when fitting the SANS data for both high and low pH. Plots are shown from (a) EDOT-FFF, Pre-GdL (Hollow cylinder model); (b) EDOT-GFF, Pre-GdL (Flexible elliptical cylinder model): (c) EDOT-GFFD, Pre-GdL (Flexible elliptical cylinder model); (d) EDOT-FFF, Post-GdL (Hollow cylinder model); (e) EDOT-GFF, Post-GdL (Flexible elliptical cylinder model); (f) EDOT-GFFD, Post-GdL (Flexible elliptical cylinder model).

Table S1. All parameters extracted from fitting of small-angle neutron scattering data. The * highlights the large errors found when fitting EDOT-GFF before gelation as mentioned in the main text.

Compound	Model	Kuhn length (Å)	Inner radius (Å)	Outer radius (Å)	Axis ratio	χ2
**Pre-GdL**						
EDOT-FFF	Hollow cylinder	n/a	6.79 ± 1.47	31.46 ± 3.4	n/a	1.81
EDOT-GFF	Flexible elliptical cylinder	122.7 ± 11.7	n/a	4.94 ± 9282*	1.00 ± 3757*	16.1
EDOT-GFFD	Flexible elliptical cylinder	151.2 ± 32.2	n/a	26.8 ± 1.7	2.53 ± 0.39	2.21
**Post-GdL**						
EDOT-FFF	Hollow cylinder	n/a	9.21 ± 0.5	32.3 ± 3.4	n/a	3.04
	Flexible cylinder	433.04 ± 27.56	n/a	33.71 ± 0.16	n/a	2.52
EDOT-GFF	Flexible elliptical cylinder	85.5 ± 20.5	n/a	40.9 ± 2.1	2.5 ± 0.1	2.93
EDOT-GFFD	Flexible elliptical cylinder	327.6 ± 20.1	n/a	22.9 ± 0.5	2.6 ± 0.1	1.04

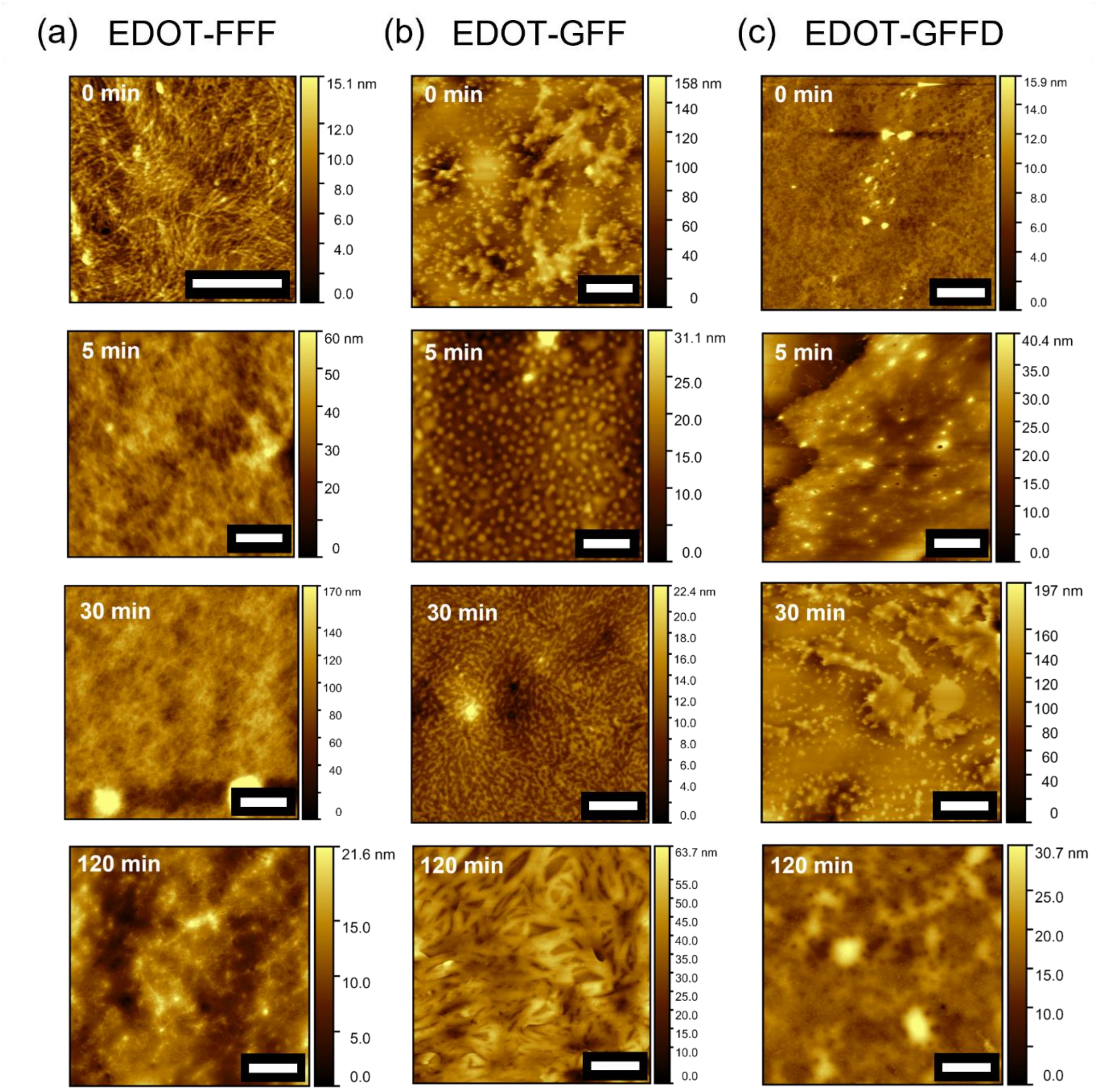

Figure S5. AFM images taken over the course of gelation for the 3 gelators (a) EDOT-FFF; (b) EDOT-GFF; (c) EDOT-GFFD. Scale bars: 2 μm.

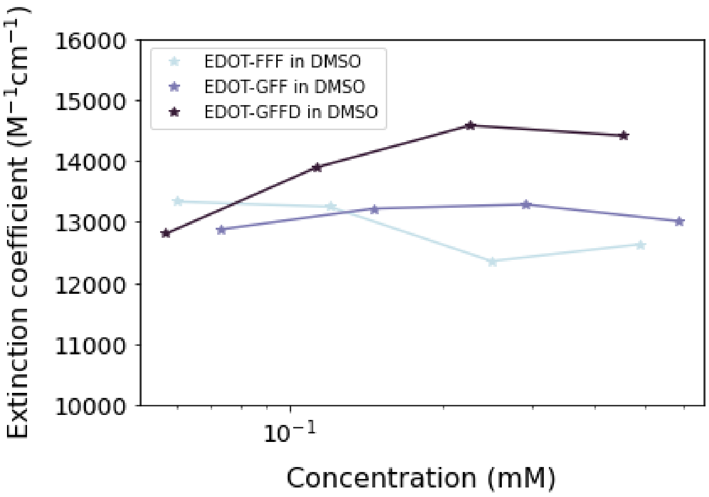

Figure S6. Extinction coefficient of the three gelators in DMSO with dilution.

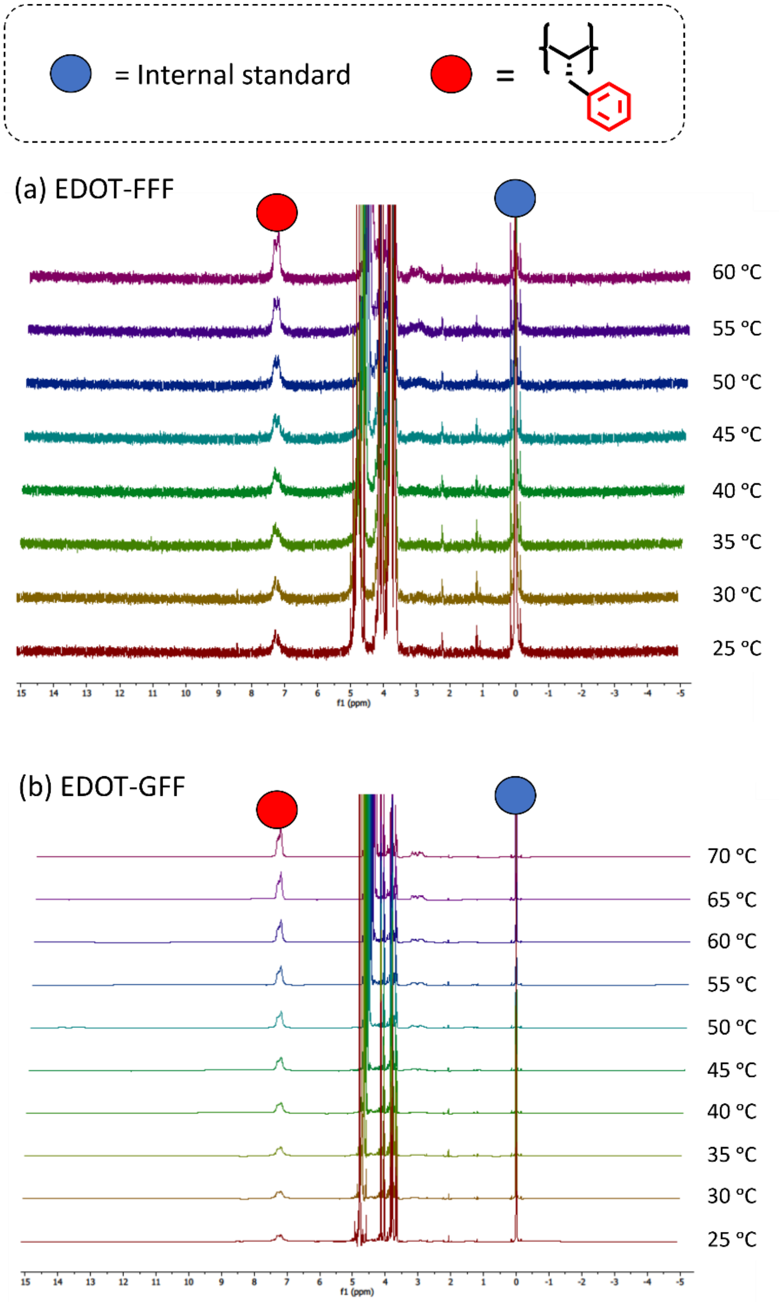
= Internal standard
=
(a) EDOT-FFF
60 °C
55 °C
50 °C
45 °C
40 °C
35 °C
30 °C
25 °C
f1 (ppm)
(b) EDOT-GFF
70 °C
65 °C
60 °C
55 °C
50 °C
45 °C
40 °C
35 °C
30 °C
25 °C
f1 (ppm)

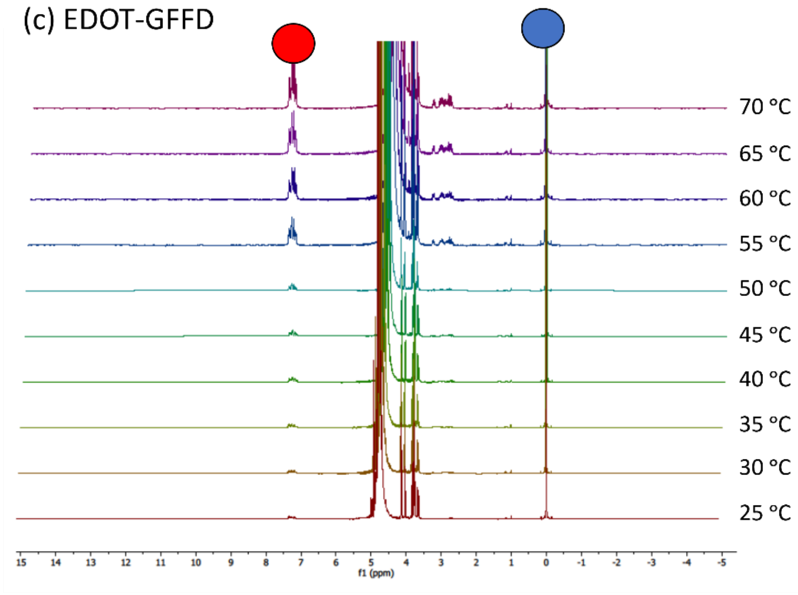

Figure S7. Stacked proton NMR spectra of (a) EDOT-FFF; (b) EDOT-GFF and (c) EDOT-GFFD. The peaks highlighted by the red circle come from the phenyl protons of the phenylalanine amino acids in each compound respectively. An internal standard of 3-(trimethylsilyl)propionic-2,2,3,3-d_4 acid sodium salt was used to calculate the concentration of these phenyl protons and thus of the compounds at increasing temperatures.

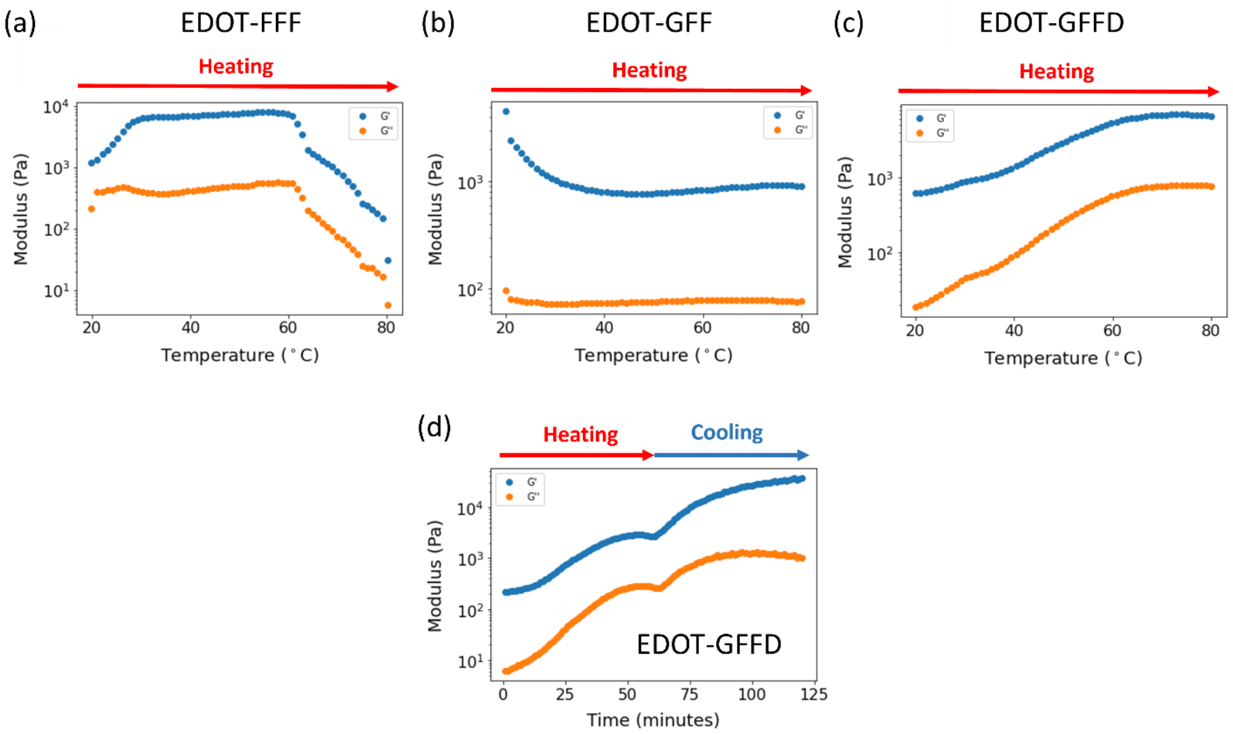

Figure S8. Rheology data showing the storage and loss moduli for (a) EDOT-FFF; (b) EDOT-GFF and (c) EDOT-GFFD with increasing temperature from 20-80 °C post-gelation. In (d) a heating and cooling cycle from 20 to 80 °C and back again, 1 degree per minute is shown for EDOT-GFFD i.e. at the 60 minute mark, the temperature is at 80 °C and begins to decrease again. All gels at a concentration of 10 mM.

Note in the case of EDOT-FFF, the gel begins to lose mechanical integrity from 60 °C, as such NMR data was not considered above this temperature when estimating thermodynamic dissociation constants. Of particular interest is the apparent thermal toughening of EDOT-GFFD up to 70 °C.

In **Figure S8d** is shown the full heating and cooling cycle for EDOT-GFFD. Here can be seen a change of 2 orders of magnitude in storage modulus as mentioned in the main text.

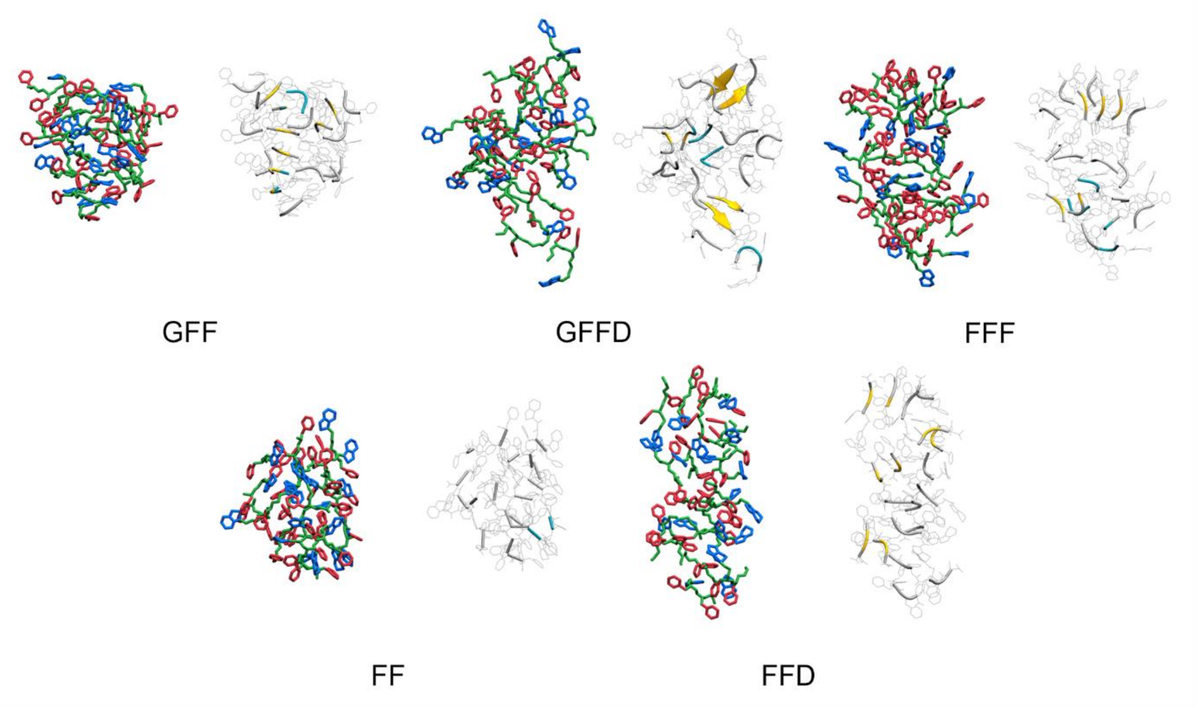

Figure S9. Representative self-assembled oligomer structures of twenty EDOT-GFF, EDOT-GFFD, EDOT-FFF, EDOT-FF, and EDOT-FFD peptides after 100 ns of MD simulations to highlight the solvent exposed residues and predominant interactions. For each peptide sequence, the left image shows the (blue) EDOT moiety, (red) aromatic side-chain groups, and (green) hydrophilic peptide backbone respectively, hydrogen atoms not shown. The right image depicts the peptide's secondary structure characteristics, to emphasise the emerging beta character observed for EDOT-GFF and EDOT-GFFD made possible by the flexible glycine linker compared to EDOT-FFF, EDOT-FF, and EDOT-FFD. Beta turns shown as yellow lines and beta sheets shown as yellow arrows. EDOT and aromatic residues shown as thin grey lines with coils shown in silver and turns in cyan. Solvent molecules, hydrogen atoms, and periodic unit cells not displayed for clarity.

Table S2. Newly defined CHARMM-compatible parameters (in GROMACS format) for EDOT-peptides

```
[ bondtypes ]
;      i        j  func           b0           kb
  CG2R51   OG3R60  1  0.13650000  254815.64 ; EDOT
  CG2R51   OG3R60  1  0.13650000  254815.64 ; EDOT-PEP
   CG2O1    CG2O5  1  0.15100000  215911.14 ; EDOT-PEP
   CG2O1      NH1  1  0.13450000  309616.00 ; EDOT-PEP

[ angletypes ]
;      i        j        k  func       theta0       ktheta          ub0          kub
   CG321    CG321   OG3R60  5  111.500000 376.560000 0.00000000       0.00 ; EDOT
  CG2R51   OG3R60    CG321  5  108.803000 625.641888 0.00000000       0.00 ; EDOT
  CG2R51   CG2R51   OG3R60  5  174.966000 160.104944 0.00000000       0.00 ; EDOT
   CG2O5   CG2R51   SG2R50  5  124.270000  78.441632 0.00000000       0.00 ; EDOT-PEP
  CG2R51   CG2R51   OG3R60  5  174.966000 160.104944 0.00000000       0.00 ; EDOT-PEP
  CG2R51   OG3R60    CG321  5  108.803000 625.641888 0.00000000       0.00 ; EDOT-PEP
   CG321    CG321   OG3R60  5  111.500000 376.560000 0.00000000       0.00 ; EDOT-PEP
   CG2O1    CG2O5   CG2R51  5  107.150000 323.339520 0.00000000       0.00 ; EDOT-PEP
   CG2O1    CG2O5    OG2D3  5  116.550000 751.781120 0.00000000       0.00 ; EDOT-PEP
   CG2O5    CG2O1    OG2D1  5  118.032000 870.054432 0.00000000       0.00 ; EDOT-PEP
   CG2O5    CG2O1      NH1  5  109.059000 488.942240 0.00000000       0.00 ; EDOT-PEP
   OG2D1    CG2O1      NH1  5  122.500000 669.440000 0.00000000       0.00 ; EDOT-PEP
   CG2O1      NH1        H  5  123.000000 284.512000 0.00000000       0.00 ; EDOT-PEP
   CG2O1      NH1      CT2  5  120.000000 418.400000 0.00000000       0.00 ; EDOT-PEP
   CG2O1      NH1      CT1  5  120.000000 418.400000 0.00000000       0.00 ; EDOT-PEP (PHE)

[ dihedraltypes ]
;      i        j        k        l  func         phi0         kphi  mult
  OG3R60    CG321    CG321   OG3R60  9     0.000     0.815880 3 ; EDOT
  OG3R60    CG321    CG321     HGA2  9     0.000     0.815880 3 ; EDOT
  OG3R60   CG2R51   CG2R51   SG2R50  9   180.000    33.342296 2 ; EDOT
    HGA2    CG321   OG3R60   CG2R51  9     0.000     3.845096 3 ; EDOT
   CG321    CG321   OG3R60   CG2R51  9   180.000    13.777912 1 ; EDOT
   CG321    CG321   OG3R60   CG2R51  9     0.000     9.790560 2 ; EDOT
   CG321    CG321   OG3R60   CG2R51  9   180.000    11.719384 3 ; EDOT
  OG3R60   CG2R51   CG2R51   OG3R60  9     0.000     8.598120 2 ; EDOT
  CG2R51   CG2R51   OG3R60    CG321  9     0.000     0.255224 2 ; EDOT
  CG2R51   CG2R51   OG3R60    CG321  9   180.000     1.435112 4 ; EDOT
  OG3R60   CG2R51   CG2R51    HGR52  9     0.000    12.627312 2 ; EDOT
  CG2R51   CG2R51   CG2R51   OG3R60  9   180.000     4.171448 2 ; EDOT
   CG2O5   CG2R51   SG2R50   CG2R51  9   180.000    11.242408 2 ; EDOT-PEP
   CG2O5   CG2R51   CG2R51   OG3R60  9   180.000     1.719624 2 ; EDOT-PEP
   OG2D3    CG2O5   CG2R51   SG2R50  9   180.000     5.108664 2 ; EDOT-PEP
   CG2O1    CG2O5   CG2R51   SG2R50  9   180.000    25.710680 2 ; EDOT-PEP
   CG2O1    CG2O5   CG2R51   CG2R51  9   180.000     0.091630 2 ; EDOT-PEP
   OG2D1    CG2O1    CG2O5   CG2R51  9   180.000    17.355232 2 ; EDOT-PEP
     NH1    CG2O1    CG2O5   CG2R51  9     0.000    14.932696 2 ; EDOT-PEP
   OG2D1    CG2O1    CG2O5    OG2D3  9   180.000    23.045472 2 ; EDOT-PEP
     NH1    CG2O1    CG2O5    OG2D3  9     0.000     1.891168 2 ; EDOT-PEP
   CG2O5    CG2O1      NH1        H  9   180.000     9.740352 2 ; EDOT-PEP
   CG2O5    CG2O1      NH1      CT2  9   180.000     1.631760 1 ; EDOT-PEP
   CG2O5    CG2O1      NH1      CT2  9   180.000     1.096208 2 ; EDOT-PEP
   OG2D1    CG2O1      NH1        H  9   180.000    10.460000 2 ; EDOT-PEP
   OG2D1    CG2O1      NH1      CT2  9   180.000    10.460000 2 ; EDOT-PEP
   CG2O1      NH1      CT2      HB2  9     0.000     0.000000 1 ; EDOT-PEP
   CG2O1      NH1      CT2        C  9   180.000     0.836800 1 ; EDOT-PEP
   OG2D1    CG2O1      NH1      CT1  9   180.000    10.460000 2 ; EDOT-PEP (PHE)
   CG2O1      NH1      CT1        C  9   180.000     0.836800 1 ; EDOT-PEP (PHE)
   CG2O1      NH1      CT1      HB1  9     0.000     0.000000 1 ; EDOT-PEP (PHE)
   CG2O1      NH1      CT1      CT2  9     0.000     7.531200 1 ; EDOT-PEP (PHE)
   CG2O5    CG2O1      NH1      CT1  9   180.000     1.631760 1 ; EDOT-PEP (PHE)
   CG2O5    CG2O1      NH1      CT1  9   180.000     1.096208 2 ; EDOT-PEP (PHE)

[ dihedraltypes ]
;      i        j        k        l  func         phi0         kphi
   CG2O5    CG2O1   CG2R51    OG2D3  2     0.000   602.496000 ; EDOT-PEP
   CG2O1    CG2O5      NH1    OG2D1  2     0.000  1004.160000 ; EDOT-PEP
```

Table S3. GROMACS topology with 22ptimized atom charges and atom types for the EDOT-dicarbonyl fragment

```
[ EDTL ] ; EDOT connected to dicarbonyl linker (i.e. EDOT-COCO-PEP)
  [ atoms ]
        S    SG2R50 -0.1210   1
      CA1    CG2R51  0.0000   1
      CB1    CG2R51 -0.0020   2
      OG1    OG3R60 -0.2950   2
      CD1     CG321  0.1170   2
     HD11      HGA2  0.0900   2
     HD12      HGA2  0.0900   2
      CD2     CG321  0.1170   3
     HD21      HGA2  0.0900   3
     HD22      HGA2  0.0900   3
      OG2    OG3R60 -0.2950   3
      CB2    CG2R51 -0.0020   3
      CA2    CG2R51 -0.1320   1
      HA2     HGR52  0.2280   1
       CX     CG2O5  0.0450   1
       OX     OG2D3 -0.3410   1
        C     CG2O1  0.6770   1
        O     OG2D1 -0.3560   1
  [ bonds ]
       S    CA2
       S    CA1
     CA1    CB1
     CA1     CX
     CB1    CB2
     CB1    OG1
     OG1    CD1
     CD1   HD11
     CD1   HD12
     CD1    CD2
     CD2   HD22
     CD2   HD21
     CD2    OG2
     OG2    CB2
     CB2    CA2
     CA2    HA2
      CX     OX
      CX      C
       C      O
       C     +N
  [ impropers ]
      OX    CA1     C     CX
```

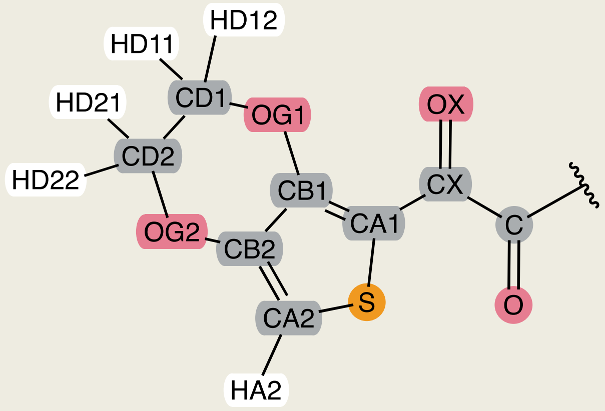

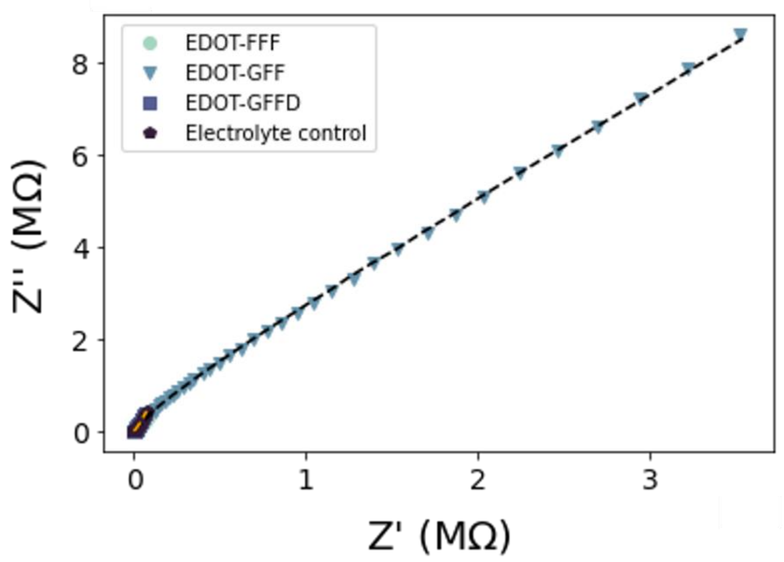

Figure S10. Full impedance spectra of the three gelators and electrolyte control showing how EDOT-GFF gave a much bigger impedance magnitude when compared to the other gelators. Fits shown by dashed lines, electrolyte control fit shown by an orange dashed line, though difficult to observe give difference in Impedance magnitude between EDOT-GFF and the other samples.

Table S4. Parameters generated through modelling of the mid-to-low frequency impedance data. EDOT-FFF and EDOT-GFF are modelled using the circuit shown in Figure 7b and EDOT-GFFD and the electrolyte control are modelled using the circuit shown in Figure 7a.

Compound	R_{sol} (Ω)	W_O-R (Ω $s^{-1/2}$)	W_O-T ($s^{1/2}$)	CPE_1-T ($\Omega^{-1}s^{CPE1-P}$)	CPE_1-P	CPE_2-T ($\Omega^{-1}s^{CPE1-P}$)	CPE_2-P	χ2
EDOT-FFF	1473 ± 6.3	2162 ± 261	$4.7x10^{-3}$ ± $3.5x10^{-4}$	$2.3x10^{-5}$ ± $2.1x10^{-6}$	0.45 ± 0.013	$1.7x10^{-6}$ ± $2.6x10^{-7}$	0.76 ± 0.034	$7.1x10^{-4}$
EDOT-GFF	2692 ± 13	881 ± 53	$7.4x10^{-5}$ ± $3.7x10^{-6}$	$3.4x10^{-7}$ ± $1.5x10^{-8}$	0.84 ± $2.5x10^{-3}$	$2.1x10^{-7}$ ± $8.7x10^{-9}$	0.57 ± $7.5x10^{-3}$	$6.8x10^{-5}$
EDOT-GFFD	2850 ± 2.5	309 ± 17	$1.6x10^{-3}$ ± $9.4x10^{-5}$	$1.1x10^{-5}$ ± $7.8x10^{-8}$	0.67 ± $1x10^{-3}$	n/a	n/a	$6.3x10^{-5}$
Electrolyte control	1856 ± 2	992 ± 28	$6.5x10^{-3}$ ± $2.5x10^{-4}$	$7.1x10^{-6}$ ± $9.1x10^{-8}$	0.75 ± $2.2x10^{-3}$	n/a	n/a	$9.97x10^{-5}$

NMR spectra

EDOT-FF: ^{1}H NMR

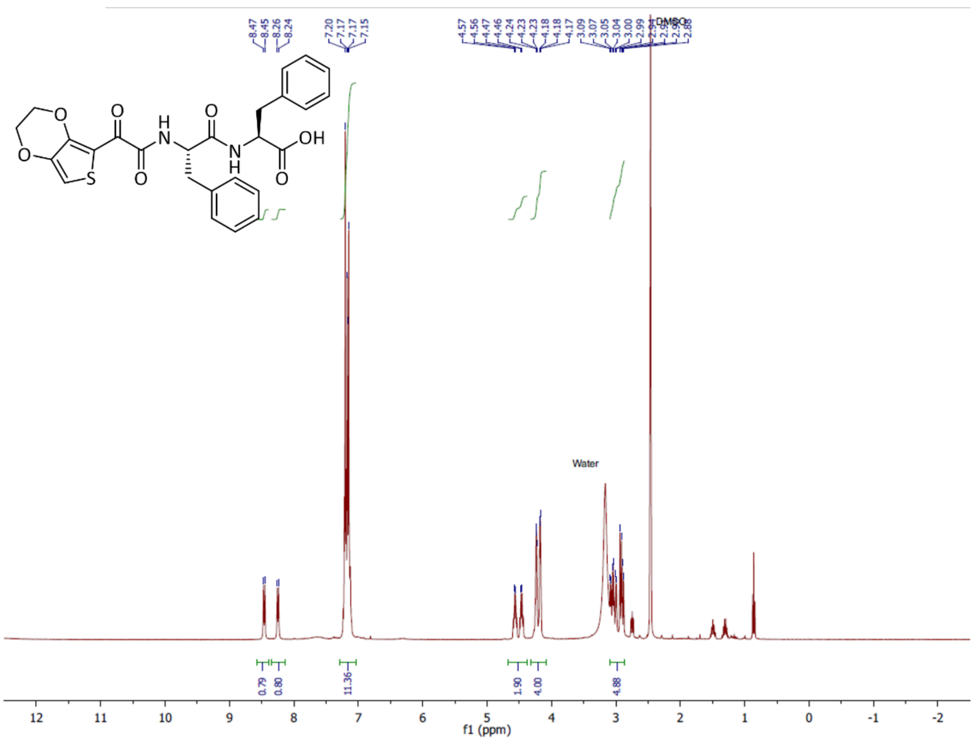

EDOT-FF: ^{13}C NMR

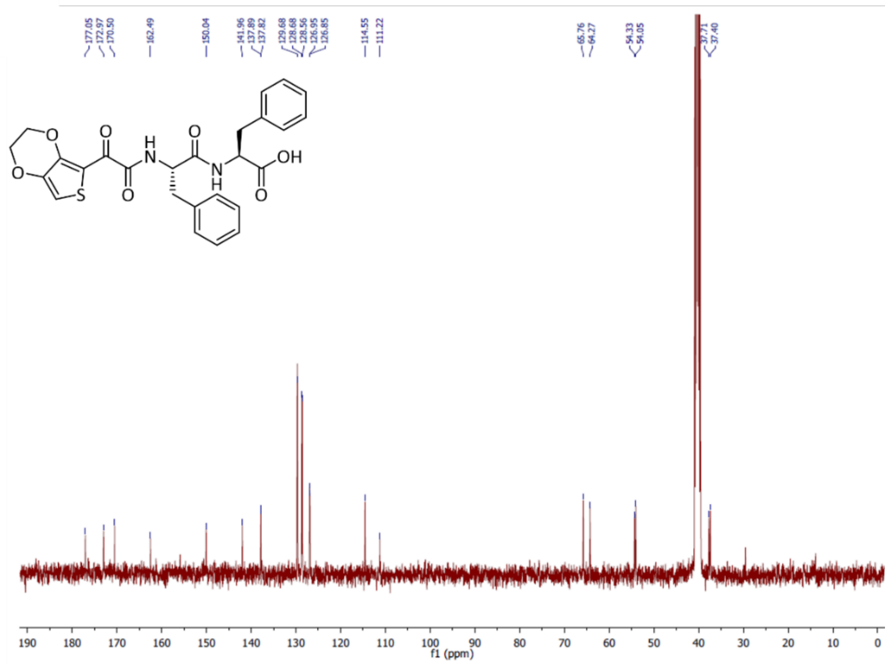

EDOT-FFD: ^{1}H NMR

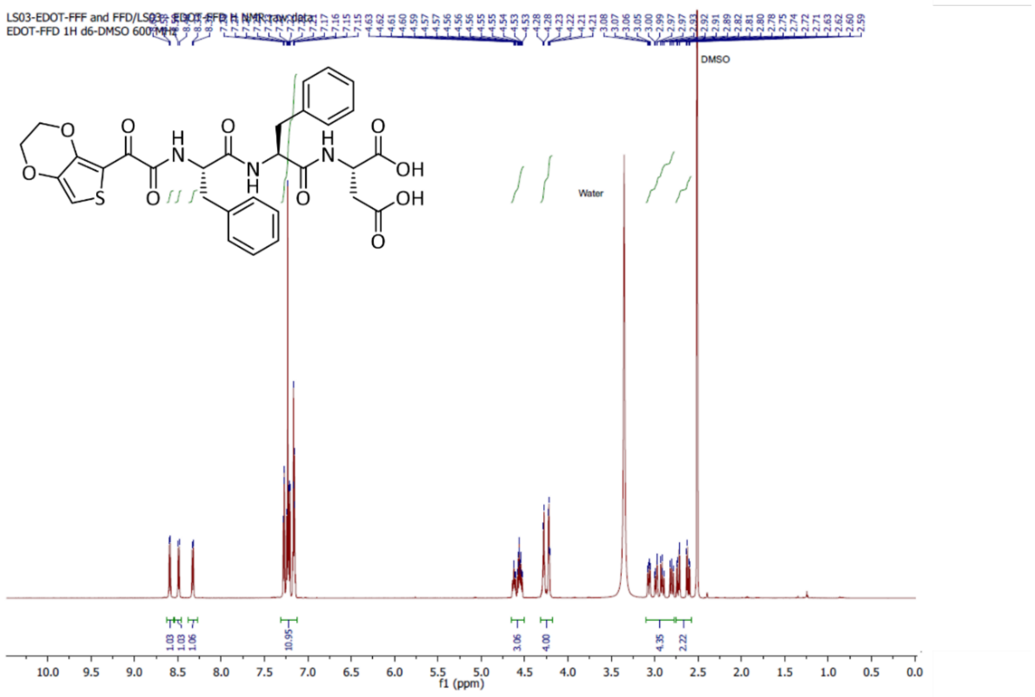

EDOT-FFD: ^{13}C NMR

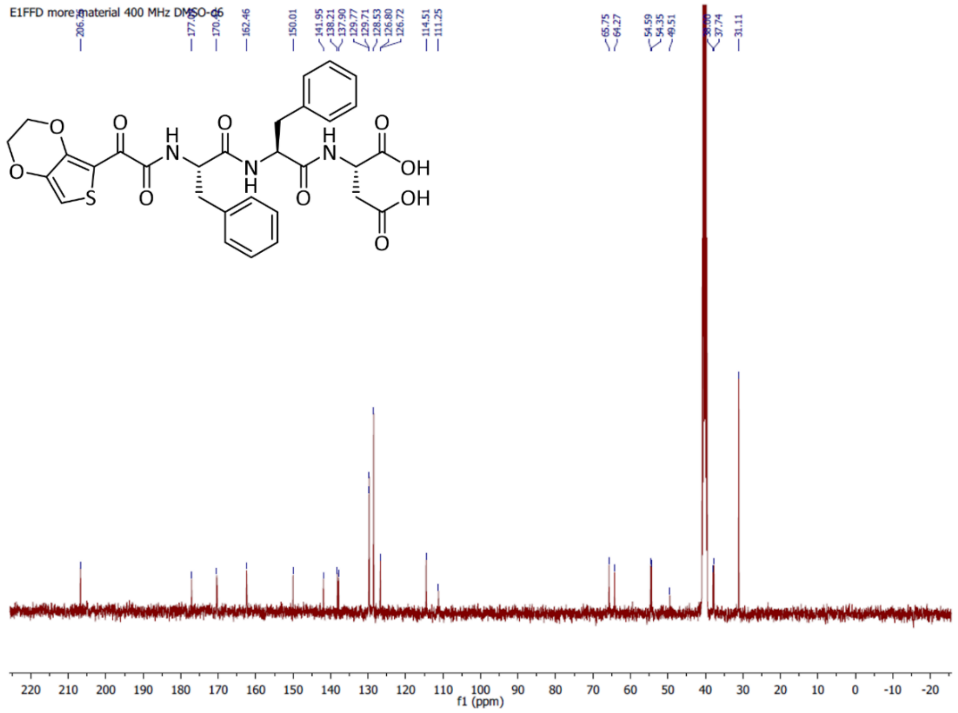

EDOT-GFF: ^{1}H NMR

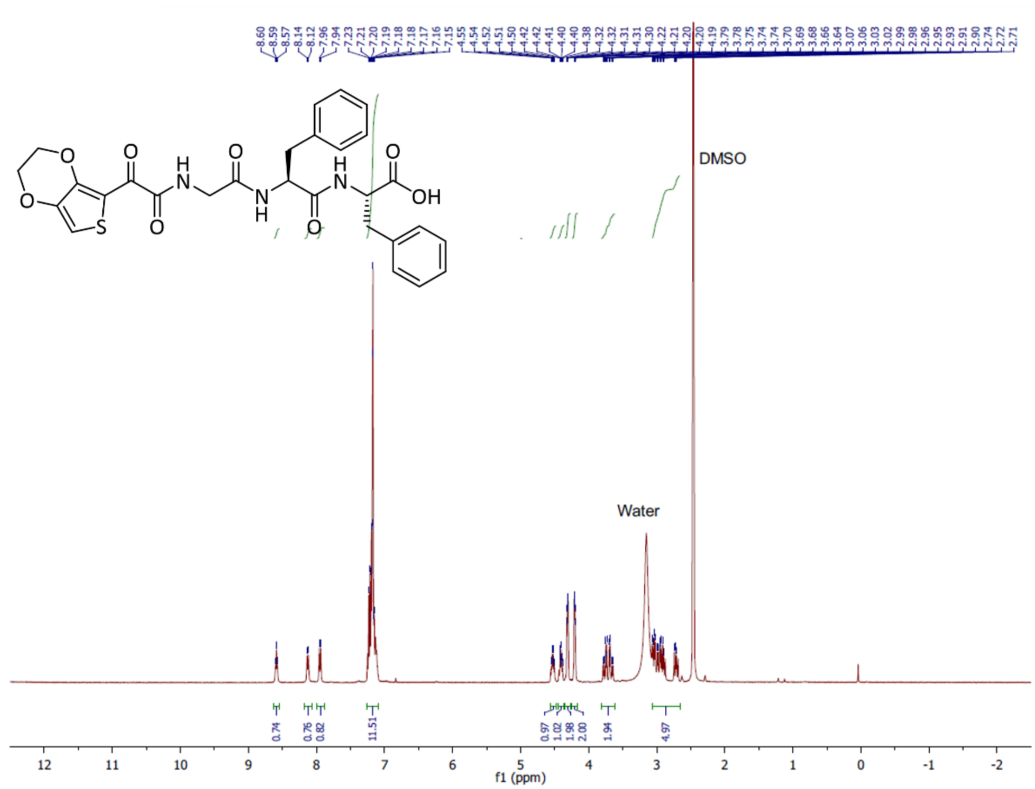

EDOT-GFF: ^{13}C NMR

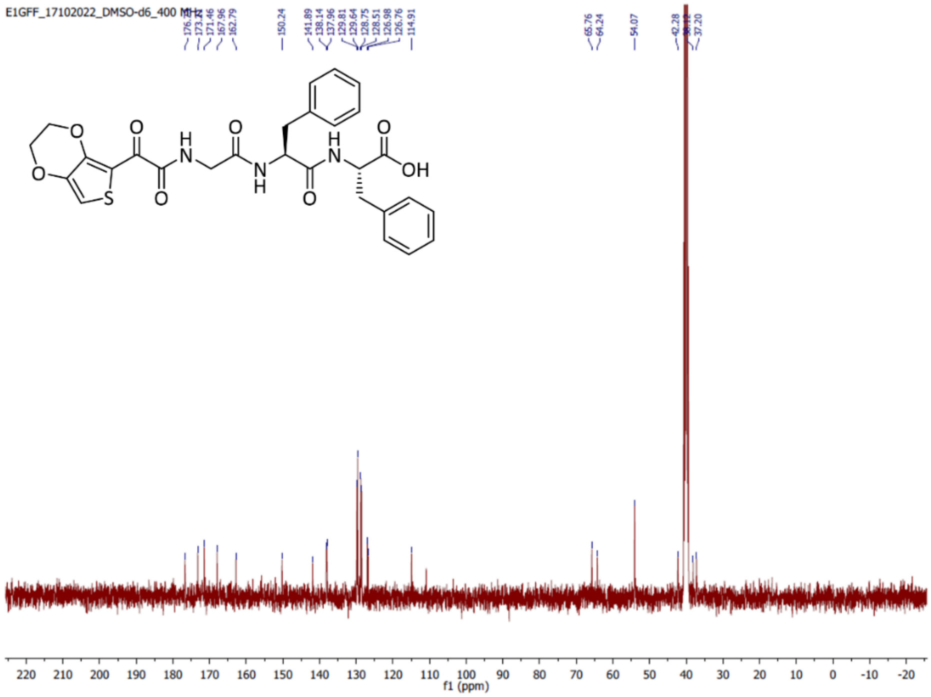

EDOT-GFFD: ^{1}H NMR

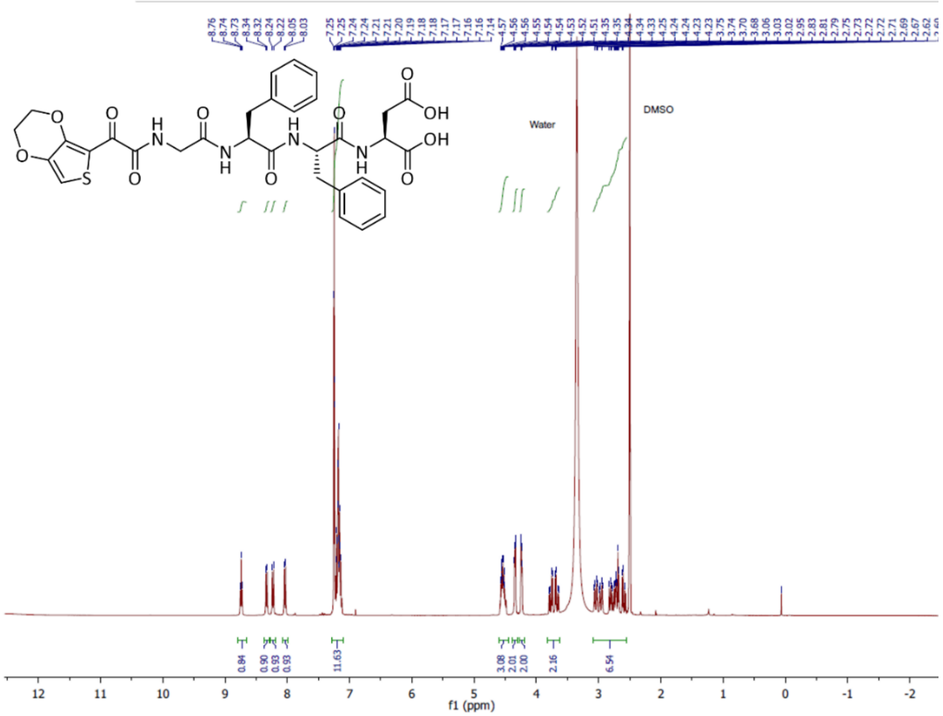

EDOT-GFFD: ^{13}C NMR

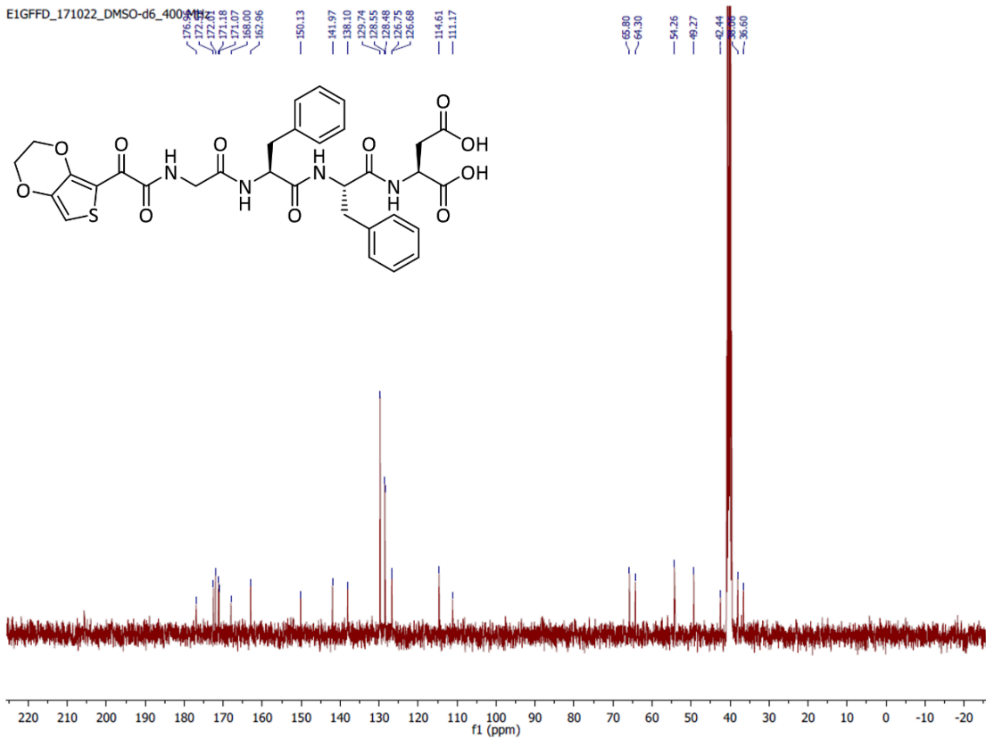

EDOT-FFF: ^{1}H NMR

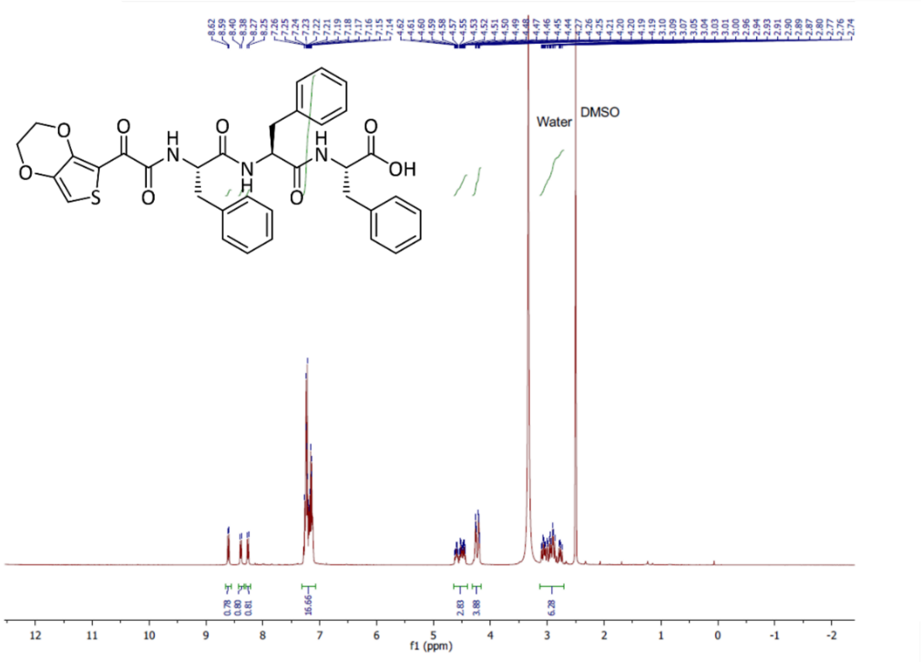

EDOT-FFF: ^{13}C NMR

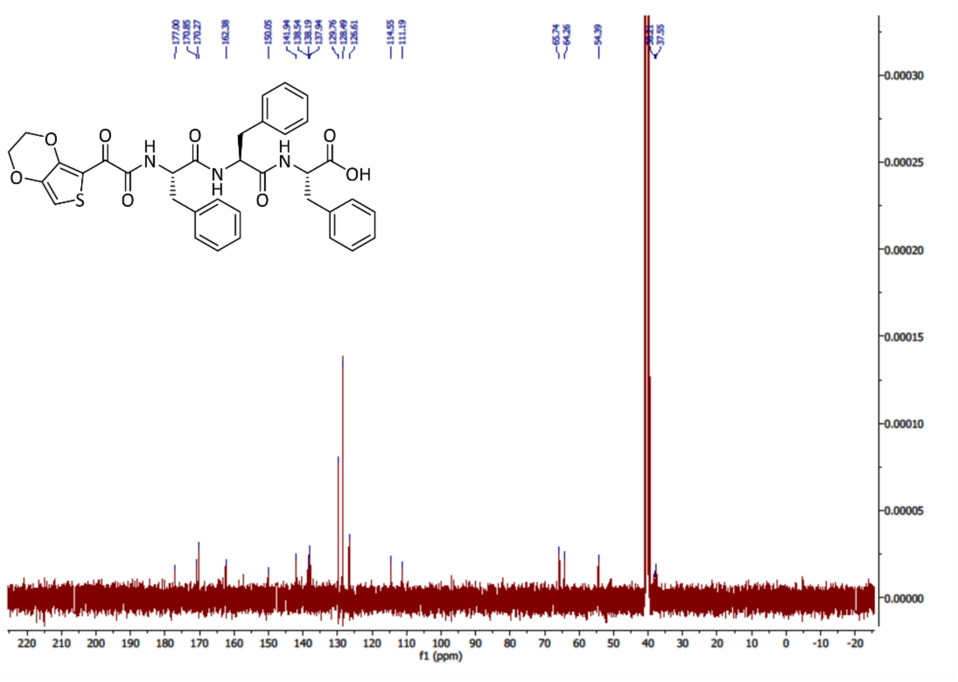

EDOT-COOH (**2**): ^{1}H NMR

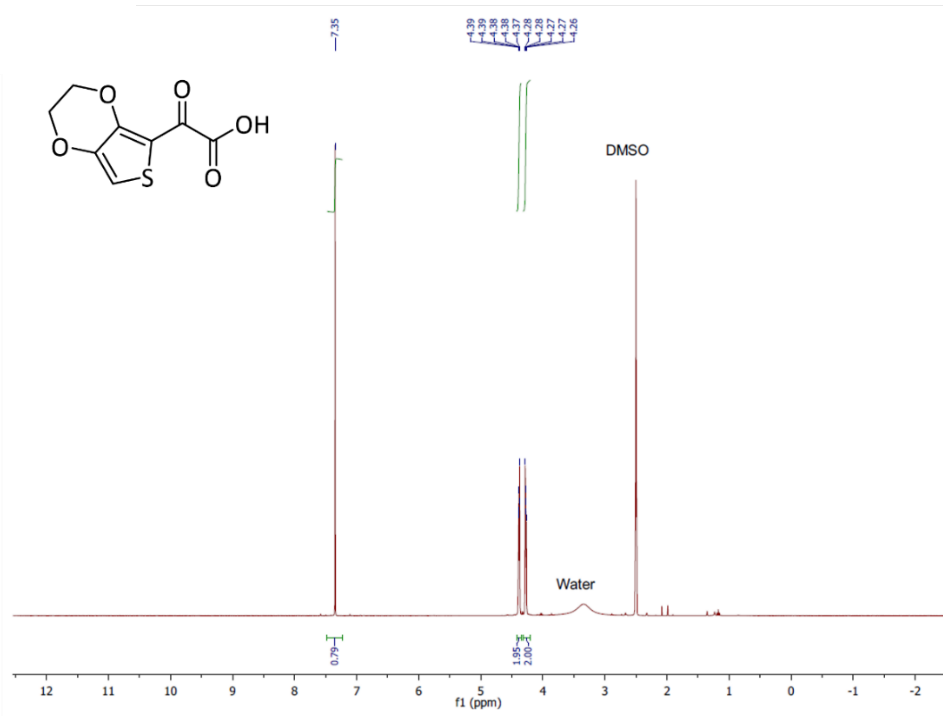

Fmoc-GFF: ^{1}H NMR

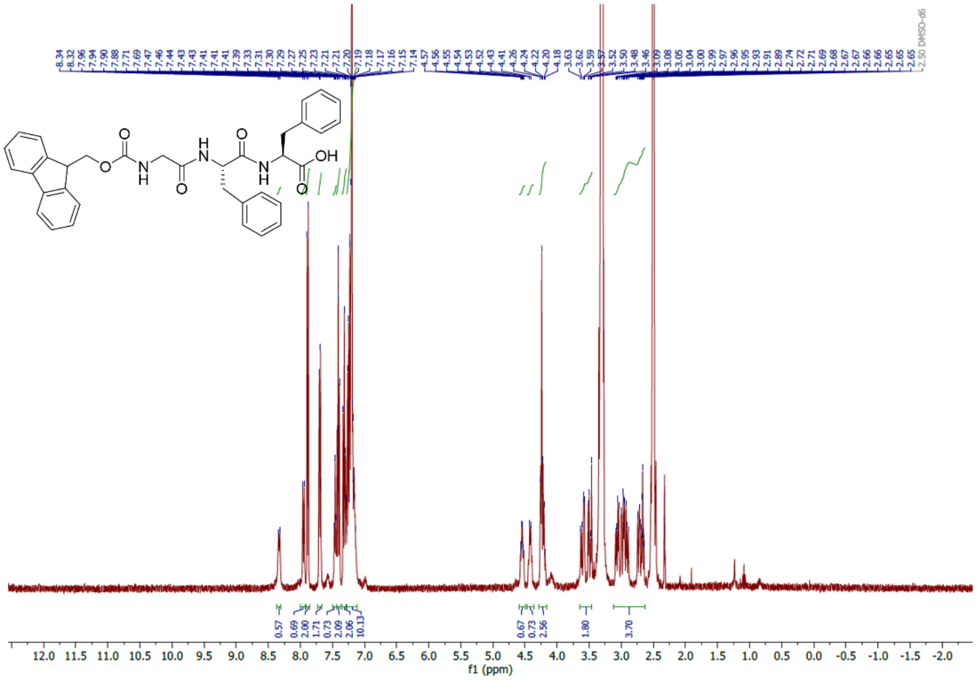

References

1. Spicer, C. D.; Booth, M.; Mawad, D.; Armgarth, A.; Nielsen, C.; Stevens, M. M. Synthesis of Hetero-bifunctional, End-Capped Oligo-EDOT Derivatives. *Chem* **2**, 125–138 (2017).

2. Wojciechowski, J. P.; Martin, A.; Mason, A.; Fife, C.; Sagnella S.; Kavallaris, M.; Thordarson, P. Choice of Capping Group in Tripeptide Hydrogels Influences Viability in the Three- Dimensional Cell Culture of Tumor Spheroids. *ChemPlusChem* **82**, 383–389 (2017).
